# Impact of technical, patient-related and measurement variables on serial Hounsfield unit–based quantitative coronary plaque analysis in computed tomography: time for a new chapter

**DOI:** 10.1093/ehjimp/qyaf014

**Published:** 2025-01-29

**Authors:** Francesca Calicchio, Elizabeth Epstein, Melinda Boussoussou, Borbála Vattay, Alexander van Rosendael, Shawn Newlander, Márton Kolossváry, Bálint Szilveszter, Pál Maurovich-Horvat, Hugo Marques, Elliot McVeigh, George Wesbey

**Affiliations:** Department of Cardiology and Radiology, Scripps Clinic, 9888 Genesee Ave, La Jolla, CA 92037, USA; Department of Cardiology and Radiology, Scripps Clinic, 9888 Genesee Ave, La Jolla, CA 92037, USA; Heart and Vascular Center, Semmelweis University, 1122 Budapest, Hungary; Heart and Vascular Center, Semmelweis University, 1122 Budapest, Hungary; Department of Cardiology, Leiden University Medical Center, 2333 ZA, Leiden, the Netherlands; Department of Cardiology and Radiology, Scripps Clinic, 9888 Genesee Ave, La Jolla, CA 92037, USA; Department of Cardiology, Gottsegen National Cardiovascular Center, Haller u. 29, 1096 Budapest, Hungary; Physiological Controls Research Center, Bécsi út 96b, Óbuda University, 1034 Budapest, Hungary; Heart and Vascular Center, Semmelweis University, 1122 Budapest, Hungary; Medical Imaging Center, Semmelweis University, Üllői út 78a, 1082 Budapest, Hungary; UNICA Radiology, Hospital da Luz Lisboa, Av. Lusíada 100, Católica Medical School, 1500-650 Lisboa, Portugal; Department of Medicine, UC San Diego School of Medicine, 9500 Gilman Dr, 92093 La Jolla, CA, USA; Department of Cardiovascular Division, UC San Diego School of Medicine, 9500 Gilman Dr, 92093 La Jolla, CA, USA; Department of Radiology, UC San Diego School of Medicine, 9500 Gilman Dr, 92093 La Jolla, CA, USA; Department of Bioengineering, UC San Diego School of Medicine, 9500 Gilman Dr, 92093 La Jolla, CA, USA; Department of Cardiology and Radiology, Scripps Clinic, 9888 Genesee Ave, La Jolla, CA 92037, USA

**Keywords:** coronary CT angiography, quantitative coronary plaque analysis, low-attenuation plaque (LAP), photon-counting detector CT (PCD-CT), energy integrating detector CT (EID-CT)

## Abstract

This review article explores the challenges and controversies involved in accurately identifying and reliably quantifying coronary plaque over time through coronary computed tomography angiography (CCTA), particularly focusing on lipid-rich, low-attenuation plaques. It highlights significant variability in lipid-rich plaque measurements across studies, questioning their reliability for tracking biological plaque transformation in clinical practice. To address this issue, the review article proposes suggestions for serial CCTA plaque measurements, aiming for realistic goals for reproducible and meaningful serial plaque CCTA imaging. It also emphasizes the necessity of standardized, validated methods for quantitative plaque analysis and underscores the potential of phantom-based calibration to improve the reliability and consistency of serial plaque measurements in clinical practice.

## Graphical Abstract

**Graphical Abstract:**
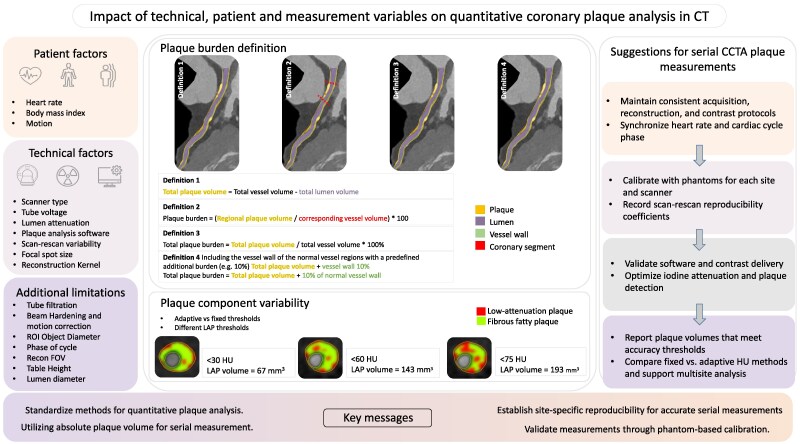

## Introduction

The results of studies showing the prognostic outcomes of coronary computed tomography angiography (CCTA) quantitative plaque analysis are encouraging; however, to date, the plaque measurements for routine clinical use between different readers, software platforms, and patient subgroups remain inconsistent, as concluded by the 2020 and 2024 Society of Cardiovascular Computed Tomography (SCCT) consensus documents.^1,2^ Serial examinations for identifying atherosclerotic progression or regression using quantitative plaque measurements require further research given the current lack of standardization of acquisition and analysis.

Computed tomography (CT) numbers are used to characterize a wide variety of normal and pathological tissues, including coronary calcium, and assess for enhancement with iodinated contrast agent.^3^ However, CT number reliability has been questioned with phantom studies since the inception of CT in the 1970s. A myriad of intrinsic patient and extrinsic technical factors can alter the CT number^4^ (*Graphical Abstract*). Materials such as iodine and calcium have vastly different chemical compositions but can share the same CT number, making it challenging to visually distinguish plaque from lumen in cardiac-gated CT. Despite the large variety of reconstruction options with modern photon-counting detector CT (PCD-CT), imaging of a commercial coronary plaque phantom with isodense calcified plaque (CP) in a 400 Hounsfield unit (HU) iodine lumen required adaptive thresholding software at 150 keV virtual monoenergetic image (VMI) to quantitate the lumen diameter.^5^ The variation in calibration factors between scanners,^6^ across patients of different sizes,^7^ and even within the same patient means that a given CT number (e.g. 130 HU) does not consistently represent the same amount of calcium.^7^ CT number will change as a function of the size of the measured object of interest,^3^ whether it be plaque^8,9^ or lumen.^10^ Using the latest PCD-CT software, a 2-mm, 5-mg/mL iodine insert in an anthropomorphic CT phantom at 65 keV showed kernel-dependent deviations from −90 to −124 HU from the nominal ground truth 147 HU.^11^ Larger objects had smaller deviations: 5  (0 to −34 HU), 1 (0 to −17 HU), and 22 mm (0 to −3 HU).^12^ Rybertt *et al*.^13^ similarly concluded that PCD-CT spectral results were highly accurate for vessels *larger* than 4 mm in diameter and regions of interest larger than 3 mm. The bias in iodine quantification increased with smaller diameters. Comparing phantom diameters from 4 to 12 mm in a PCD-CT 3D print calcified vessel wall with an iodine lumen, Liu *et al*.^14^ concluded that stable quantification of HU and iodine concentration in 70 keV virtual monochromatic images and iodine density maps was achieved at lumen diameters *greater than 6 mm*. Since quantitative coronary plaque analysis of much smaller regions of interest uses absolute CT numbers as a proxy for material composition, it becomes necessary to use phantoms as a reference standard for the quality assurance of the HU itself. This function cannot be provided by intravascular ultrasound (IVUS) or histology alone. Healy *et al*.^15^ combined histology ground truth *and* phantom calibration (calcium chloride) for spectral material identification of lipid and water and calcium with spectral PCD-CT.

## Aim of the review

Quantitative plaque analysis and serial plaque assessment are complex fields with several variables related to factors such as different CT scanners, contrast media, scanning protocols, image reconstruction, and plaque analysis software. This review delves into the challenges associated with the limited spatial resolution present in today's conventional energy-integrating detector CT (EID-CT) scanners and the improvements in spatial and spectral resolution offered by PCD-CT.

We attempt to address the questions: what proof is needed in future *serial* CCTA plaque analysis studies to establish adequate evidence that proves biological (rather than technical) change in coronary plaques? What is the scan–rescan variability for all plaque compositional measurements? Does the claimed therapeutic effect account for this variability? We particularly focus on lipid-rich low-attenuation plaque (LAP), because of the established vulnerability to rupture leading to acute coronary events.^1^ The rich wealth of knowledge and the prominent role of phantoms in the many decades of non-contrast CT coronary calcium are reviewed in Supplementary data online, *Appendix Section S1*.

We review the discrepancies and challenges of this intricate topic and discuss the rationale for phantom-based calibration solutions. Phantoms provide reliable and quantitative data inherent within medical images.^3,5,8–10,16,17^ Most importantly, these measurements can be directly traceable to the national or international technical standards [e.g. units of the International System of Units (SI)], ensuring an unbroken chain of comparisons with recognized references.^18^ The CT number, (HU), is not an SI unit, such as milligrams or cubic millimetres. Commercially available anthropomorphic phantoms can play a crucial role in calibrating and comparing imaging systems as first demonstrated in the landmark coronary calcium CT 2007 multi-vendor consortium.^7^ The Agatston *score* is a mathematical construct, and there is no physical reference standard against which to compare. It does not correspond to any physical unit of measurement of calcium. Both Agatston and conventional volume scores exhibit high variability within and between different CT systems, affecting risk assessment.^19^ The consortium proved the calcium *mass* measurement as the most accurate and reproducible measurement. Rather than using a fixed HU value of 130 for calcium, the CT number was *adaptively* adjusted based on the phantom measured *calibration factor* to correspond to 100 mg/cm^3^ calcium hydroxyapatite (HA) for each scanner and protocol and body size.^7^ Using this HA density threshold yielded very consistent results for mass measurements across small, medium, and large thorax diameters. This same phantom-based standardized calibration can be used in theory to reproducibly identify and quantify a clinically important substance [e.g. cholesterol and cholesteryl esters in lipid-rich necrotic core (NC) plaque] in a volume of interest. The Radiological Society of North America's Quantitative Imaging Biomarkers Alliance^20^ and the European Society of Radiology's European Imaging Biomarkers Alliance,^21^ the Image Biomarker Standardization Initiative,^22^ and other international organizations all share a common goal for quantitative imaging: *reproducibility*. CCTA plaque volume measurements need to have maximal reproducibility across imaging centres, imaging equipment, participants, body sizes, and time.^23^

We focus primarily on HU-based methods. Several other approaches include machine learning, combinations of multiple measurements, or spectral CT analysis.

## The 2020 and 2024 SCCT plaque expert consensus documents: a crucial starting point

**Figure 1:**
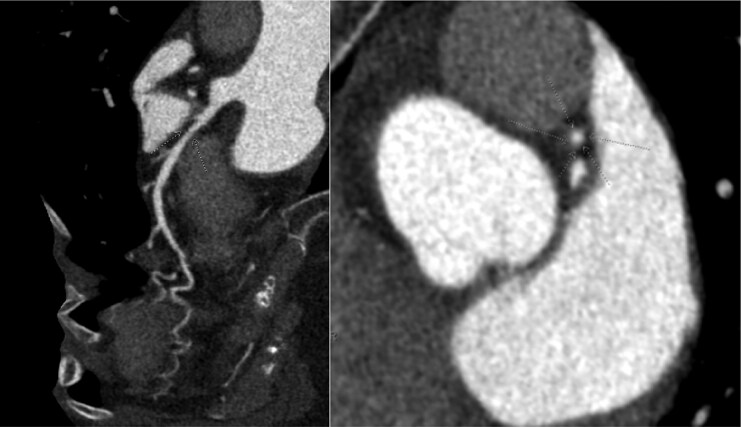
NCP in the proximal left anterior descending artery (arrows).

The SCCT expert consensus documents review the quality of evidence for CCTA plaque assessment.^1,2^ One of the conclusions is that ‘evidence is valid and highly correlated with invasive or pathologic measures and there is high accuracy for detection of at-risk patients.^1^  *Figure 1* shows an example of commercial plaque analysis software identification of a small (11.8 mm^3^) non-calcified plaque (NCP) in the proximal left anterior descending (LAD) artery in a CCTA study read as normal by a Level 3 reader.

However, the consensus concludes that plaque cannot be reliably measured among different patients’ subgroups, interpreting physicians and software platforms. Wide variations in minimum lumen area and total and compositional plaque volume were shown among seven vendors in an example analysing the exact same single proximal segment of the LAD.^2^ It underlines how serial CCTA plaque measurements for identifying atherosclerosis progression are not clinically established and should be considered only for research purposes at this time. Similarly, a review by Williams *et al*.^24^ concluded that, currently, the proof of research is inadequate to endorse clinical serial CCTA plaque measurements.

Although plaque analysis can predict outcomes, several problems with serial CCTA plaque measurements need to be addressed, the greatest of which include limited spatial resolution and partial volume averaging. Numerous additional factors^24–26^ are summarized in the *Graphical Abstract*. No standard vocabulary for plaque measurement output exists, particularly for low attenuation plaque (LAP). Williams *et al*.^24^ recommend discarding dependence on IVUS and histology and suggest standardized phantom-based models and clinical studies establishing similarities and differences between different quantitative software to enable reproducibility among vendors. We also encourage standardization through this approach.

**Figure 2:**
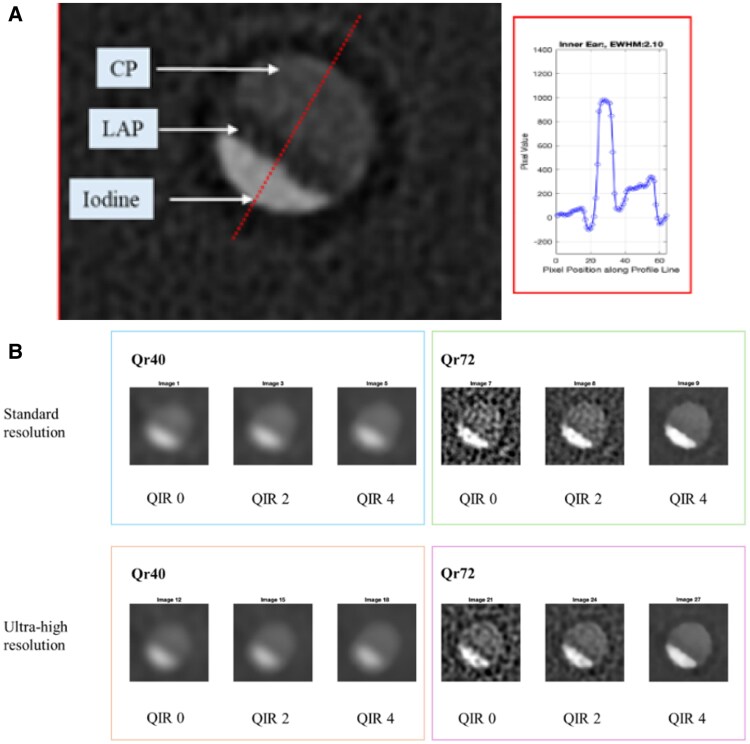
(*A*) Ground truth ultra-high-resolution mode with inner ear PCD-CT non-gated protocol, excellent visualization of +75 HU plaque with no increase in low attenuation plaque attenuation. (*B*) Representative enlarged images of gated PCD-CT of the plaque phantom feature using both softer and sharper kernel settings and different levels of QIR. Clear visual detection of the central LAP feature is limited to UHR-Qr72, but attenuation is increased from +75to +175 HU. The increase is even higher for other reconstructions.

Phantoms can be designed with specific biologically relevant synthetic components to mimic the CT relevant attenuation. The material attenuation properties depend on (i) X-ray absorption and (ii) scattering. This review aims to extend these recommendations with illustrative commercially available phantoms^5,27^ and patient^28^ examples and stimulate further research into the optimal phantom design,^8,9,16,29^ such as dual-filament 3D printing for CT scanners using materials such as calcium-based polylactic acid with StoneFil (FormFutura).^13,14,30^ We have demonstrated in a mixed plaque phantom the extreme challenges even PCD-CT faces associated with the detection of 1-mm thick 75 HU plaque interposed between 1-mm thick 1000 HU iodine lumen attenuation and 2-mm thick 300 HU calcific plaque.^31^ High lumen attenuation increases the +75 HU plaque to 175–400 HU depending on acquisition and reconstruction details (*Figure 2A*).

## Review of CCTA methodology for the measurement of coronary plaque volumes

### HU-based methods

The CCTA plaque measurements that correlated the HU value with the plaque type were the first logical choice because of their ease of use and ubiquitous availability on all CT scanners.^32^ Initial research showed a lower HU value for lipid-rich plaques than for fibrous plaques. However, a significant HU measurement overlap and inter-observer variability were reported between soft and fibrous plaques at a single tube voltage.^33^ The beam-hardening effects from iodine and calcium (*Figure 2B*)^31^ can hinder precise measurement of the HU value of plaques, resulting in overlap of HU in lipid-rich and fibrous plaques.

Analysis can provide volumetric cubic millimetres (mm^3^) for coronary plaque and lumen assessments. These values are sometimes normalized to the total vessel volume or the volume of a specific segment, expressed as a percentage. Alternatively, mm^3^ can be normalized to the total plaque volume (TPV) or total NCP volume (NCPV) in the coronary tree. The denominator varies, including the total vessel volume or length, segment vessel volume or length, and TPV or total NCPV.^34^ Some software only considers the vessel volume in segments with plaque. Normalized measures, such as per cent atheroma volume (PAV), aim for consistency with IVUS atheroma measurement; however, the range of denominators can be confusing. In addition, some techniques validating histology or IVUS report the square millimetre (mm^2^)^35^ surface area of plaque rather than the mm^3^. The same coronary segments need to be measured in serial scans to avoid a spurious change due to total segment number mismatch. Plaque burden is inconsistently defined; the lumen volume can easily change as a function of vasodilator variability, image quality, and the phase of the cardiac cycle;^36^ and it is not always possible to match systolic or diastolic phases on serial scans. Uncertainty about the start and end of a specific vascular segment will introduce variation in the measurement, and the PAV values normalized by segment will generally be much higher. It is also sometimes challenging to determine whether a given study used a lesion-based approach without contouring the normal segments or added a plaque burden to the normal vessel parts. The total vessel volume is often measured for the entire coronary vessel up to a certain lower diameter lower limit (i.e. 2.0 mm) to recognize the reality of partial volume averaging for vessel diameter under 2.0 mm.^10^ The total vessel volume depends on the patient body surface area and is more pertinent with low total vessel volumes than with high total volumes. Normalization may well be more appropriate to account for risk assessment.^37^ One might ask, why do we ne to normalize cubic millimetres to any denominator for serial scans in the same individual to assess biologic change?

We propose—for the sake of standardization and uniformity—that all future *serial* plaque measurements *in an individual patient* be reported in mm^3^ over the entire measurable coronary tree. This will optimize reproducible comparisons and maintain fidelity with newer and more accurate coronary artery calcium volume units of measurement (mm^3^).^28,38^ This is the simplest metric of choice for standardization for *serial* intra-individual CCTA plaque analysis, and is produced by every commercial CCTA plaque analysis vendor.^38^ Age- and sex-specific nomograms^39^ and staging classifications should continue to be developed based on mm^3^. Because several research publications only report plaque burden without providing for the TPV or total vessel volume in mm^3^, we review both the PAV (denominator total coronary tree vessel volume) and mm^3^ throughout this study to allow appropriate comparisons.

### Fixed HU threshold

Despite being widely adopted, the fixed HU threshold has several intrinsic limitations. Many factors beyond just tube voltage and iodine delivery rate affect the measured HU attenuation of a given tissue.

For the sake of simplicity, we focus this review with designation of plaque types into three major categories: CP, NCP, and LAP (also known as LRNC). The LRNC forms due to pathological retention of lipids, particularly lipoproteins, within the intimal and medial arterial wall cells.^40^ The numerous synonymous terms for LAP/LRNC in the literature include low-density NCP (LD-NCP), NC, NC volume, low-density NC, low-attenuation NCP, NC tissue, lipid core volume, lipid-rich coronary plaque, lipid-rich, low-density plaque, low-density core, LAP volume, and soft plaque.

The proposed fixed HU threshold ranges for each of these sub-types began in 2007. The widely cited seminal studies in the CCTA field to report low-attenuation volume and NCPV with validation by IVUS was that of Motoyama *et al.*^41,42^ in 2007. They found that the greatest accuracy to IVUS was achieved with an LAP fixed threshold of +30 HU *at 135 kVp*. The ceiling cut-off for the NCP was +350 HU. Since this is an original article, various definitions of LAP using different tube voltages have been used in the literature with varying HU thresholds, e.g. for LAP, ceilings of +30, +45, +60, +75, and +90 have been used.^34,43^ An in-depth review of LAP measurements from the literature is found in Supplementary data online, *Appendix Section S2*.

Mancini *et al.*^44^ compared LAP, NCP, and CP compositional plaque volume measurements in 8 patients and 24 coronary segments at 2 different LAP fixed HU ceilings of +30 and +75 HU. For LAP, the mean focal plaque volume at +75 HU ceiling was 0.192 mm^3^/mm, whereas at +30 HU, it was 0.062 mm^3^/mm. The Bland–Altman (BA)^45^ bias of +0.131 mm^3^/mm was systematic, with a trend towards increasing difference with increasing magnitude of measurement (*r* = 0.481, *P* = 0.02). The 95% limits of agreement (LOAs) were wide as well from −0.002 to 0.264 mm^3^/mm. In contrast, NCP and CP had small differences comparing +30 and +75 HU ceilings.

The INVICTUS trial^46^ investigated artificial intelligence (AI) quantitative CT in 133 coronary plaques from 47 patients vs. reference standard near-infrared spectroscopy-IVUS (NIRS-IVUS). NIRS-IVUS detects the major lipids in LAP and focuses on overall lipid burden. The optimal HU and plaque volume thresholds for most accurate LAP quantification were LAP of <30 HU and ≥2.30 mm^3^ and remodelling index (RI) of ≥1.1, with accuracy of 94%, sensitivity of 93%, and specificity of 94%. Lumen attenuation was not reported.

### Fixed HU with PCD-CT

**Figure 3:**
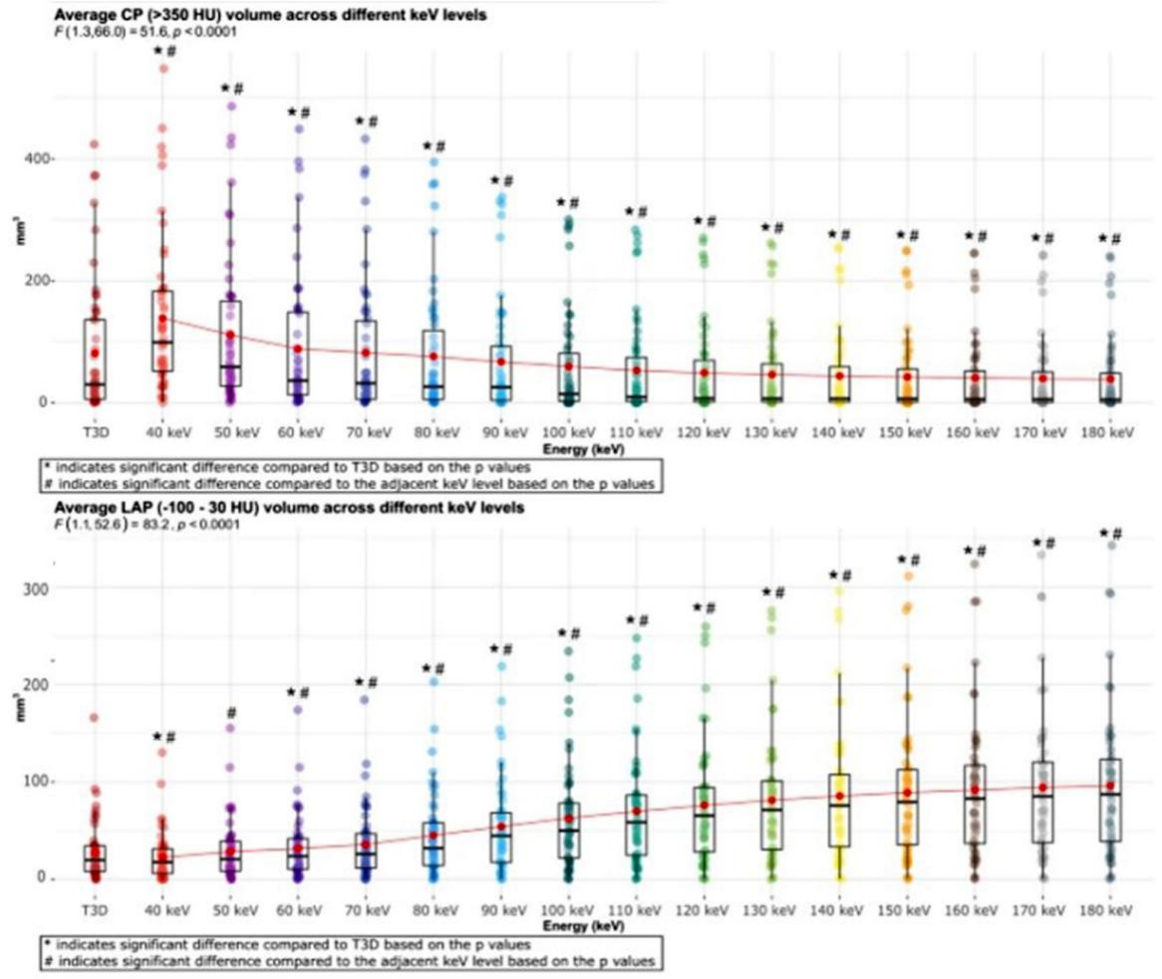
Relationship between the LAP volume, CP volume, and virtual monoenergetic energy (kiloelectron volts) in PCD-CT.^47^

**Figure 4:**
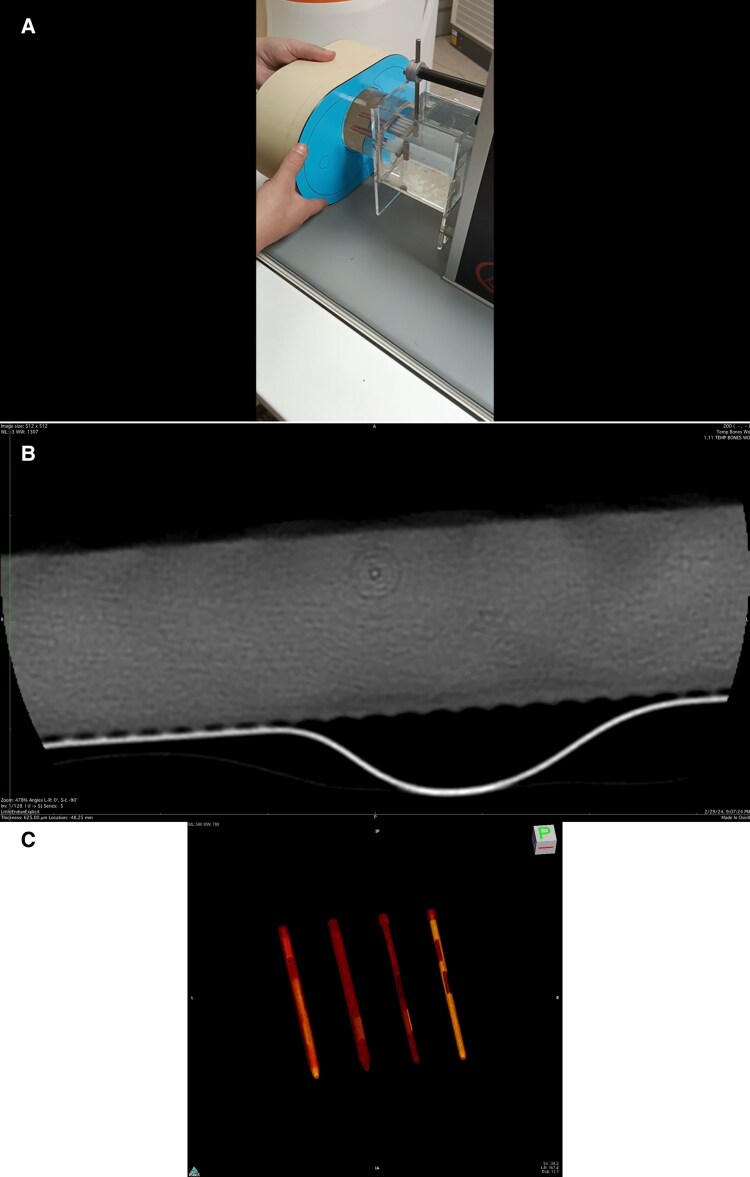
(*A*) Videoclip. Video demonstration of four commercial coronary plaque phantoms in water bath inside thorax phantom in PCD-CT system. Cardiac motion simulator at far right. (*B*) Videoclip. Same four phantoms CCTA sequential axial CT planes of sections on a 256-slice volumetric EID-CT in ground truth mode with highest available spatial resolution using full rotation temporal bone protocol. (*C*) Videoclip. Same four phantoms volume-rendered CT scans on a 256-slice volumetric EID-CT in ground truth mode with highest available full rotation spatial resolution.

**Figure 5:**
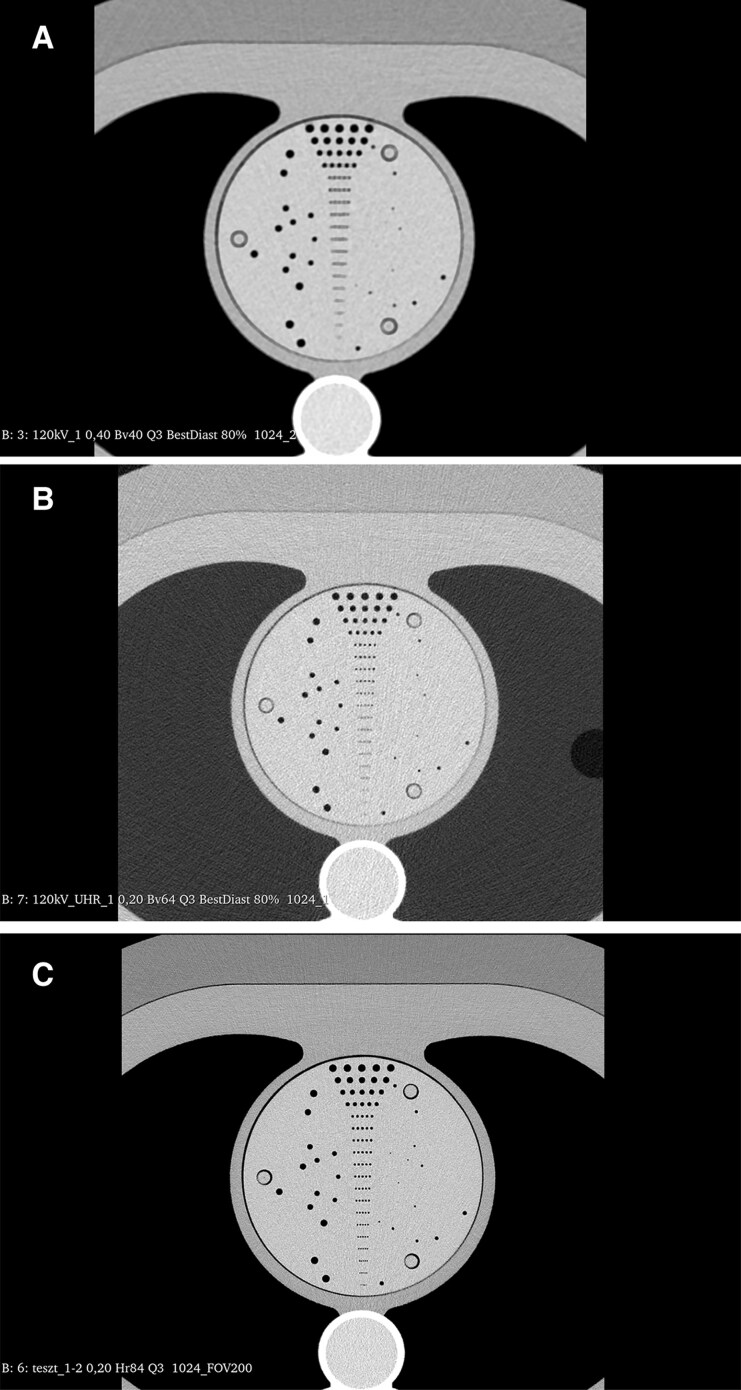
Hole phantom insert in an anthropomorphic chest phantom surrounded by 35 cm fat attenuation ring. Smallest visible hole is: (*A*) 1.5 mm for standard 0.4 mm slice thickness gated acquisition Bv40 kernel focal spot 1.0 × 1.2 mm, (*B*) 0.75 mm for UHR 0.2 mm slice thickness gated acquisition Bv64 kernel focal spot 1.0 × 1.2 mm, (*C*) 0.35 mm for the inner ear 0.2 mm slice thickness non-gated full rotation Hr84 kernel focal spot 0.7 × 0.7 mm (ground truth reference). All images 1024 × 1024 matrix.

Although +30 HU appears to be the most commonly used threshold, no study has rigorously examined the singular effect of photon energy on lumen and compositional plaque volume at a fixed ceiling of +30 HU until the PCD-CT publication by Vattay *et al.*^47^ They examined 15 different VMI reconstructions of photon energy (ranging from 40 to 180 keV) in 51 plaques from 51 patients.^47^ The VMI images are created from water-iodine material basis pair decomposition and mimic different monochromatic (single photon energy) X-ray sources.^48^ These VMI HU values are specific to the water-iodine material basis pair and would change with different material basis pairs (e.g. cholesterol-water.^49^) They differ from conventional CT image generation in EID-CT where all energies above a certain noise threshold (typically 30 keV) are integrated. PCD-CT devices directly generate a signal that measures the energy of each individual photon striking the detector.^50^ As predicted by the PARADIGM sub-study of Takagi *et al.,*^51^ the LAP volume increased, and the CP volume decreased in a nonlinear fashion with increasing photon energy (keV) (*Figure 3*). At 40 keV, LAP volume per plaque is 27.5 mm^3^ per plaque (LAP, <30 HU). It can be observed that the measured volume per plaque increases to 96.3 mm^3^ at a VMI of 180 keV. Where on the spectrum of values does the truth lie? This multi-energy analysis can be applied to plaque phantoms with known plaque volumes and attenuation to determine the optimal acquisition and reconstruction for the most accurate volume measurement of plaque type. What establishes the ground-truth volume in the phantom itself? First, the phantom manufacturer's product specifications will define the dimensions and volume of material in each synthetic plaque: lipid-rich, mixed, and calcific plaque. This is less than ideal. The better approach is the use of the exact same CT scanner's temporal bone high resolution 360° helical rotation small field of view protocol at maximum radiation exposure to establish ground truth of object dimension^48^ (*Figure 4*). We can apply spectral CT techniques (e.g. plaque cholesterol crystal EID-CT material decomposition fused in to virtual monochromatic CTA images^49^ or spectral PCD-CT) to both commercially available (*Figure 4*; see Supplementary data online, *Videos S1–S3*) and custom coronary plaque phantoms^31,52^ (*Figure 5*) with same-scanner micro-CT ground-truth determination^52^ of plaque compositional volumes. By using the same scanner's X-ray tube energy spectrum and tube filtration and imaging chain, one avoids the problems of interpreting the same measurements on a different vendor's micro-CT system with a different X-ray tube spectrum. All cardiac CT is performed with 180° rotation or less. The detector sampling frequency (views per rotation), and therefore, the spatial resolution, is reduced by 50% in cardiac CT compared with 360° helical full rotation.^53^ Other determinants of spatial resolution in CT include X-ray focal spot size, detector cell size, and convolution kernel.^10,54–56^ The fundamental limits in PCD-CT on spatial resolution of cardiac gating with reduced detector sampling and larger focal spot size (1.0 × 1.2 mm) compared with full rotation helical scan can be seen in *Figure 5*. Using visual inspection, the smallest lumen that was visualized as distinct holes with PCD-CT was (i) 1.5 mm for standard 0.4 mm slice thickness gated acquisition Bv40 kernel, (ii) 0.75 mm for ultra-high resolution (UHR) 0.2 mm slice thickness gated acquisition Bv64, and (iii) 0.35 mm for the inner ear 0.2 mm slice thickness non-gated full rotation Hr84 (ground truth reference).^57^ The gated UHR Bv64 detail resolution of 0.75 mm is a significant improvement compared with gated EID-CT detail kernel cutoff diameter of 1.3 mm.^10^ Calibration curves can be developed not only for vessel diameters^57^ but also for different material basis pairs and VMI energies. The PCD-CT calibration *combined with deep learning* can then be applied to EID-CT, similar to the concepts of Chang et al.^58^ Different *optimal* VMIs can be applied concurrently for each material of coronary plaque volume measurement to employ the advantage of each energy level: for example, 140 keV for LAP and 80 keV for CP.^48^ As Vattay et al. note, standardized acquisition protocols and multiple adaptive plaque type–specific correction factors may permit transformation between the plaque volumes from different PCD-CT VMIs and then apply to traditional EID-CT scanner images.^58^

## Need for societal guidelines and inter-vendor comparison illustrated by divergent LAP results in high-risk populations

Nurmohamed *et al.*^59^ found >15% total PAV and NCP PAV >7.5% (vessel volume denominator) were predictive of major adverse cardiovascular event (MACE) with fixed HU machine learning software with long-term MACE on a 10-year follow-up. The LAP volumes were not even reported. Based on the very low LAP volumes in the contemporary high-lumen-attenuation fixed HU literature, they were likely very low.^51,60^ The LAP discordance with the SCOT-HEART sub-study (7% LAP plaque burden in the myocardial infarction group and 4% burden in the control group)^61^ is striking and addressed by these authors:

There are a few potential reasons for this difference in plaque quantification. First, this may be because different scanning platforms were used. Second, the approach to the calculation of noncalcified and LD plaque differed between the two studies: standardized semiautomatic software versus AI-guided plaque quantification. In the absence of a head-to-head comparison of software platforms and a unified societal guideline regarding plaque quantification, whether our findings will be affirmed in other long-term follow-up cohorts when assessing both LD-NCP and NCPV remains to be determined.’

Software with adaptive scan-specific settings was used in the SCOT-HEART *post hoc* analysis.^61^ Plaque burden was defined as the volume of the given plaque component divided by the vessel volume, but only for segments with atherosclerosis, which can be interpreted as the disease burden of affected segments. However, as only affected segments are used for calculation, a patient with a single small focal lesion with relatively large plaque to vessel volume ratio in that small section will have a significantly larger plaque burden than an individual who has diffuse disease with larger overall plaque volume and number of involved coronary segments. This definition of plaque burden does not provide any information on the total amount of plaque of the patient, as the ratio only involves segments with plaque, rather than providing a metric of the overall disease burden of the patient.

In summary, the confusing array of denominators for plaque burden that are used to define *risk* do not have to be used at all in *serial plaque measurements* for an individual patient. All vendors report cubic millimetres of volume. One measurement (mm^3^) should be more reproducible than two (mm^3^/something else). As for the issue of LAP plaque HU thresholds, we concur with the conclusions of Jávorszky *et al.*^62^: ‘The wild scattering of HU thresholds likely signifies the limits of this method in the identification of low-attenuation plaques.’ The limits of the method are the use of fixed HU thresholds for LAP in a partial volume admixture of many materials in conventional polychromatic EID-CT.

## Adaptive threshold

Several sites and vendors customize the tube voltage based on body habitus to minimize radiation dose exposure. A number of studies have shown that the plaque attenuation values are profoundly influenced by lumen contrast attenuation.^51,63,64^ As shown by Takagi *et al.*,^51^ a higher lumen attenuation, whether from lower kilovoltage peak or higher iodine concentration, is associated with a decreased LAP volume and increased CP volume. Therefore, adaptive post-processing plaque analysis software has been developed to compensate for these errors.

**Figure 6:**
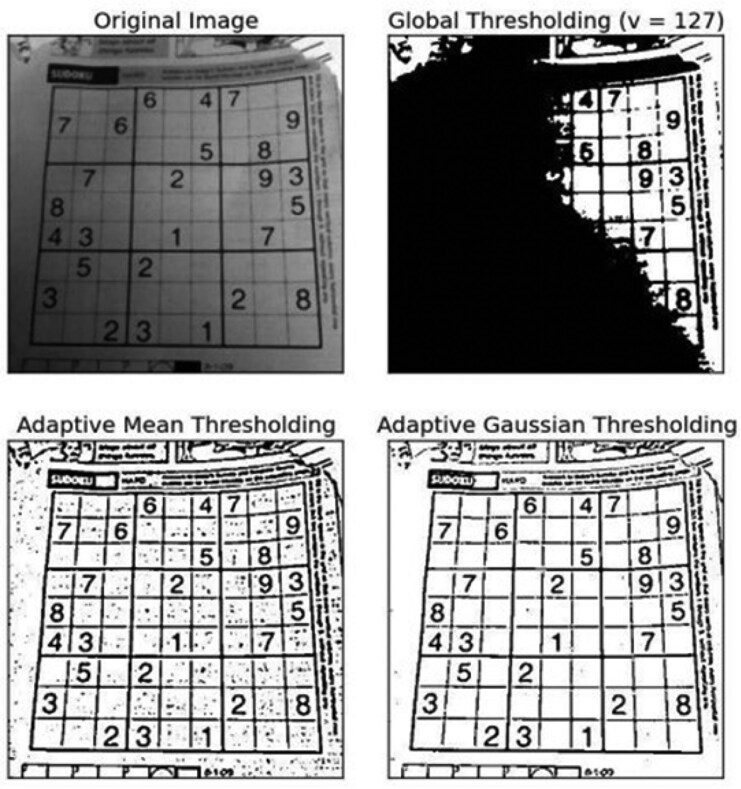
Illustration of the adaptive and Gaussian thresholding (https://docs.opencv.org/3.4/d7/d4d/tutorial_py_thresholding.html).

Adaptive thresholding adjusts visualization settings locally to enhance contrast across regions of varying densities within an image. Unlike fixed settings, an algorithm sets thresholds based on small, localized regions, allowing optimal visualization of areas with different attenuations, where calcified and soft plaque require distinct settings dependent on iodine lumen attenuation. This is illustrated in *Figure 6*.

The adaptive threshold method has shown to be less influenced by technical factors.^65^ One vendor employs scan-specific attenuation cut-offs to differentiate the lumen, NCP, and CP. It then uses knowledge-based segmentation and modelling of the coronary arteries to delineate the 3D distributions of the NCP and CP components.^66^ A large number of studies have been published with this software. Adaptive threshold algorithms were employed in the *post hoc* analysis of the landmark SCOT-HEART trial using scan-specific thresholds for plaque constituents with plaque definitions adjusted to the contrast attenuation in the aorta (this vendor's methods are reviewed in the Supplementary data online, *Appendix, Section S3*).^66^

de Graaf *et al.*^67^ proposed an adaptive threshold method where the HU thresholds are dynamically adapted according to the lumen and plaque attenuation values (see Supplementary data online, *Appendix Section S4*). They demonstrated a smaller system bias and only slight variation of plaque components compared with one with a fixed threshold.^67^ Over 100 studies have been published with this software; however, only a few have used this adaptive feature.^65,68–70^ Almost all of this vendor's studies have used fixed HU thresholds.

The ARCHITECT trial showed the effect of alirocumab on coronary plaque burden regression in 104 familial hypercholesterolaemia patients based on adaptive thresholding.^67,68^ Plaque was divided into fibrous, fibro-fatty, LAP, and CP. With this method, the LAP volumes were 42.2 and 8.2 mm^3^ before and 1 year after treatment, respectively, with a difference of 34 mm^3^. It is noteworthy to highlight how much higher these *adaptive* total coronary tree LAP values (in mm^3^) are than the *fixed* HU LAP PARADIGM sub-study (<1 mm^3^) using the same software.^68^

In 2020, Tanaka *et al*.^69^ studied coronary plaque in 70 asymptomatic diabetic patients over two years using this adaptive mode on a 320-slice CT scanner. Baseline %TPV of LAP was higher in patients without plaque progression (16.3%) than in those with progression (12.5%). These values vastly exceeded those from fixed mode in Takagi *et al.*'s^51^ PARADIGM study, and the adaptive mode was validated by IVUS virtual histology.

## Direct comparison of fixed vs. adaptive thresholds

Normalizing plaque with adaptive, as opposed to fixed, thresholds has been shown to improve the accuracy of plaque quantification.^71^ Compared with scan-specific thresholds, the fixed thresholds had a greater bias with wide BA LOA (−22.0 mm^3^ with −92.7 to 48.7 mm^3^) and an inferior correlation with IVUS (*P* < 0.001).

de Knegt *et al.*^65,70^ studied 260 individuals with symptoms of recent-onset chest pain and no evidence of acute coronary syndrome. The plaque volumes were assessed in 1161 segments. The authors assessed the correlation between the plaque volumes using the fixed and adaptive HU levels. Furthermore, the cohort was divided into tertiles of ascending aortic luminal contrast attenuation, and the plaque volume was evaluated. With the fixed HU levels, the CP volume increased with increasing levels of luminal contrast attenuation; however, this difference was not noted with the adaptive HU levels. The plaque volumes by the adaptive HU thresholds were more independent of the luminal contrast density for higher attenuation plaque than those by the fixed HU thresholds. However, the impact they found on NCP or LAP is difficult to interpret because of their use of a ceiling of +75 HU for LAP as opposed to the more commonly used +30 HU.

Mancini *et al.*^44^ compared fixed +30 HU vs. adaptive mode. For LAP, the mean focal plaque volume with adaptive was 0.148 mm^3^ per mm length of vessel segment, whereas at fixed +30 HU it was 0.062 mm^3^ per mm length of vessel segment. The BA bias of +0.086 mm^3^/mm was systematic, with a trend towards increasing difference with increasing magnitude of measurement (*r* = 0.674, *P* < 0.00001). The 95% LOA were wide from −0.064 to 0.237 mm^3^/mm. In contrast, NCP and CP had small differences.

### Inter-vendor comparisons

Quantitative plaque software comparative analysis on the same CCTA datasets is reviewed in Supplementary data online, *Appendix Section S5*.

### Hybrid plaque approach with the kilovoltage peak-adjusted contrast protocol

In 2019, Matsumoto *et al.*^72^ compared the effect of both tube voltage (100 and 120 kVp) and luminal contrast attenuation on NCPs and validated the results with IVUS. With similar luminal contrast attenuation at 100 and 120 kVp, they found that lipid-rich plaque and fibrous plaque attenuation was distinct when normalized for luminal contrast attenuation (*P* < 0.001). Based on these findings, they concluded that the plaque attenuation thresholds for NCPs should be adjusted based on luminal contrast attenuation.^72^

### Combination approach

Because plaque attenuation variability occurs with any number of technical factors, the concept of identification of more than one feature of high-risk plaque features has been analysed. Tomizawa *et al.*^73^ showed that a combination of a LAP volume of <60 HU and positive RI as continuous variables had superior diagnostic performance for the identification of optical coherence tomography-determined thin-cap fibroatheroma than did the individual presence of napkin ring sign or a positive remodeling (PR) of >1.1 or LAP volume of <30 HU.

### Scan–rescan variability studies for CCTA plaque measurements

**Figure 7:**
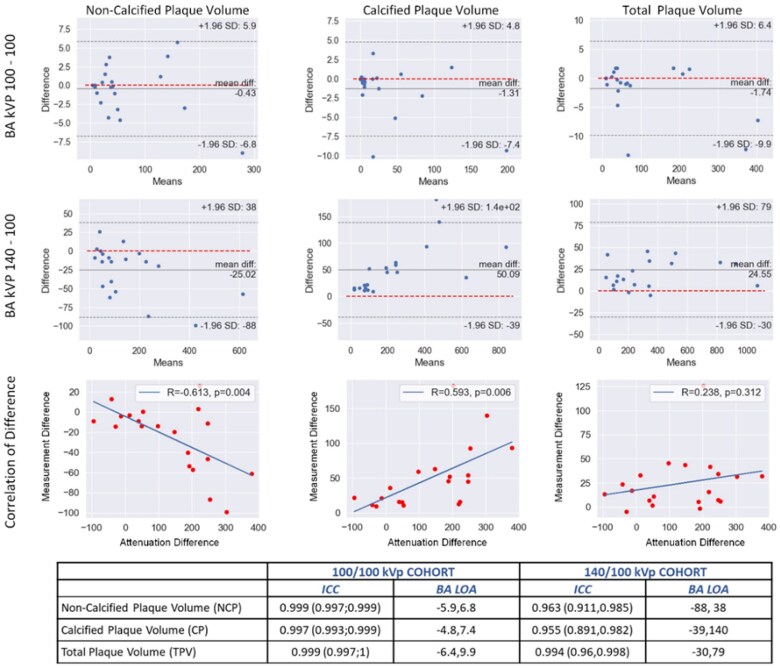
Top panel: BA and correlation plots for NCP, CP, and TPV in the 100/100 kVp vs. the 140/100 kVp group (per-patient analyses). Fixed HU threshold was used: LAP < 30 HU, NCP 30-350 HU, CP > 350 HU. Bottom panel: ICC and BA LOA for NCP, CP, and TPV. Adapted from Calicchio.^24^

We conducted a retrospective study using Food and Drug Administration–approved machine learning analysis on a 256-slice volumetric single heartbeat EID-CT scanner investigating the effect of different tube voltages (140 and 100 kVp) on plaque components in a cohort of *n* = 20 (140/100 kVp cohort) and *n* = 21 (100/100 kVp cohort) patients who underwent two scans two heartbeats apart (all other technical settings identical.^74^ Increasing ascending aortic HU difference driven by the tube voltage change between the 140 kVp (mean 520 HU) and 100 kVp (mean 653 HU) scans correlated with continuous NCP decrease (*r* = −0.61, *P* = 0.004) and CP increase (*r* = 0.59, *P* = 0.006) on linear regression analysis. The LAP values were too low to allow statistically meaningful comparisons. In the 100/100 kVp group, contrary to the 140 vs. 100 kVp group, we demonstrated that all plaque and stenosis measurements remain unchanged with >90% lower BA LOA and elimination of bias (*Figure 7*). The 100 kVp Scan 1 to Scan 2 the intraclass correlation (ICC) was 0.999, 0.997, and 0.999 for TPV, NCP, and CP, respectively. Focusing on NCP, mean bias was −25 mm^3^ for 140/100 vs. 0 mm^3^ for 100/100. NCP repeatability coefficients (smallest measurable difference)^75^ were 89 and 9 mm^3^, respectively. For this scanner's 100 kVp CCTA protocol and this plaque analysis software vendor, a change of NCP of >9 mm^3^ would represent true biological change.

An in-depth review of many additional scan-rescan CCTA plaque analysis studies is found in Supplementary data online, *Appendix Section S6*.

## Non-HU techniques for plaque quantitative analysis

### Machine learning: non-HU techniques

In 2023, Buckler *et al.*^76^ compared machine learning software with histological ground truth on sections of atherosclerotic carotid plaques from patients who underwent carotid CTA and subsequent endarterectomy. Comparing the cross-sectional area measurements of plaque constituents (mm^2^) in pre-operative carotid endarterectomy patients with specimen histology, they found machine learning software trained on carotid histological ground-truth tissue to be highly accurate in identifying carotid plaque stability phenotypes compared with expert pathologists.

### Radiomics

Beyond simple volumetric estimates of plaque components, AI techniques such as radiomics can be used to quantify the morphology and texture of lesions.^77,78^ Using predefined mathematical formulas the latent morphological characteristics of lesions can be estimated.^79^ This allows monitoring morphological changes beyond volumetric changes of coronary atherosclerosis.^80^ All radiomic variables are affected by the same patient and technical factors that affect HU-based plaque characterization, showing the need for standardization before being implemented into clinical practice.^81^

## Conclusion

We reviewed the ‘adequacy of proof’ in the published literature to date since the publication of the review by Williams *et al.*^24^ and the 2024 SCCT expert consensus document.^81,82^ A wide divergence of measured LAP volumes in mm^3^ is present across the studies. The widespread use of low tube voltages and higher iodine concentrations with fixed HU analysis has widened the LAP scatter even further. The low LAP volumes with wide scan–rescan reproducibility LOAs will not be statistically meaningful in serial studies of LAP transformation. Even with adaptive software, its users conclude that clinical trials for plaque change should focus on NCP and not LAP.

**Figure 8:**
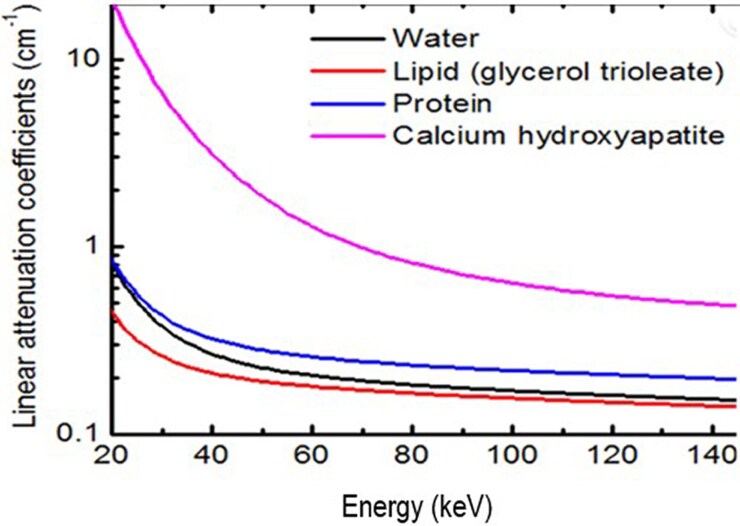
The linear attenuation coefficient (logarithmic *y*-axis co-ordinate) of biological materials as a function of X-ray energy.^67^

Have quantitative serial measurements of compositional coronary plaque volume crossed the threshold from clinical research to validated clinical practice? For those sites that have assessed scan–rescan variability for each plaque component and can report the reproducibility of their site's measurements to distinguish technical change from biologic change, an evidence base for the total plaque (TP), CP, and NCPV does exist. To the best of our knowledge, only two studies^82,83^ have reported the 95% BA confidence limits for LAP. It is time to start all over with the measurement of LAP and focus on optimization of the *acquisition* side of the CT scanner,^73^ as opposed to the *analysis* side. The quality of analysis vendor output is dependent on the quality of the acquisition input. Nothing has changed for EID-CT with fixed-HU analysis since the seminal study by Cademartiri *et al.*^64^ from long ago. Limited spatial resolution and single photon energies result in overlap of HU values in lipid rich and fibrous plaque LAP analysis. Much more sophisticated acquisition can be performed beyond single energy EID-CT. Spectral CT (*Figure 8*) with both EID-CT and PCD-CT is briefly reviewed in Supplementary data online, *Appendix Section S7*.

Our contributions/suggestions for serial CCTA plaque measurements are found in *Table 1*. The gaps in knowledge with suggestions for further research are summarized in *Table 2*.

**Table 1:** Our contributions/suggestions for serial CCTA plaque measurements

Realistic goals for reproducible and meaningful *serial* plaque coronary CT angiography imaging include the following: Report serial compositional plaque measurements *in an individual patient* in mm^3^ over the entire coronary tree Rigid adherence to constant technique for *every* technical acquisition and reconstruction parameter on serial CCTA Match heart rate and phase of cardiac cycle Site-specific, scanner-specific, and protocol-specific documentation of scan–rescan reproducibility coefficients^68^ for all plaque compositional volumes in order to establish the smallest real difference and properly detect true biologic change Best effort to match lumen iodine attenuation contrast protocols on follow-up studies Avoid different analysis vendor comparison on serial studies For LAP analysis with *fixed* +30 HU ceiling, report only individual plaque volume greater than or equal to a minimum floor of accuracy and reproducibility established by analysis vendors (e.g. INVICTUS floor 2.3 mm^3^)^69^

**Table 2:** Gaps in knowledge/suggestions for further research

The cornerstone frame of reference for EID-CT plaque and lumen phantom calibration standards should be the kVp and coronary lumen attenuation of the original publications by Motoyama *et al*.^41,42^: 135 kVp and 258 HU. These studies established the LAP ceiling of +30 HU. All compositional plaque measurements with different kVps can potentially be calibrated for this specific iodine concentration/kVp/attenuation cornerstone Different concentrations of lumen iodine can then be further calibrated from commercially available phantoms^72^ High lumen attenuation has a detrimental effect on detection of LAP.^49^ Re-examine the use of vintage low iodine delivery rate protocols for optimal LAP identification and assess impact on accuracy of stenosis quantification, particularly in heavily calcified coronary arteries and obese patients Further validate the work of Matsumoto *et al*.^72^ tailoring contrast delivery rates, volumes, and concentrations to kVp to achieve constant lumen attenuation and normalizing plaque attenuation to lumen attenuation (hybrid approach) Compare fixed HU vs. adaptive HU scan-specific analysis software with phantoms and patient data^28,38,44,65^ Ground truth for dimensions of phantom targets of interest to be established by maximum radiation and highest spatial resolution full-rotation acquisitions^31^ Ground truth for HU CT number in VMI spectral CT for phantom materials of interest (e.g. iodine, cholesterol) to be established by NIST database^11,12^ Encourage phantom-calibrated multi-site registry analysis using the exact same scanner vendor and technical and IV contrast protocols for multiple sites Optimize development of phantoms containing stenoses from NCP, which mimic the specific X-ray absorption and scattering of lipid-rich NC components of interest: LDL-cholesterol, cholesterol, triglycerides, cholesteryl esters, etc.^49^ Define image quality standards such as maximum allowable noise ceilings and minimum measurable object diameter to define the limits for plaque volume and stenosis accuracy and reproducibility in both phantoms and patients for each scanner's CCTA protocol

In this review, we have proposed and discussed rationale for site-specific, scanner-specific, and protocol-specific calibration of plaque volume in cubic mm using on site phantoms and patient data to establish reproducibility of plaque volume to best evaluate for true biologic changes taking place on serial examinations. This will require close cooperation between cardiac CT physicians and technologists, medical physicists, engineering, and material scientists, as well as co-operation with both CT vendors and software analysis vendors.

## Supplementary data

Supplementary data are available at *European Heart Journal - Imaging Methods and Practice* online.

## Supplementary Material

qyaf014_Supplementary_Data

## Data Availability

The data that support the findings of this study are available on request from the corresponding author.

## Lead author biography

Dr Francesca Calicchio stands as a distinguished cardiologist, holding licences in Italy and the UK. She completed her medical education and cardiology training at the University of Pisa, Italy, followed by training at the Royal Brompton Hospital in London, UK. In 2023, she attained her PhD in ‘Clinical Pathophysiology of Cardiovascular Diseases’ from the University of Pisa in close collaboration with the Lundquist Institute at Harbor-UCLA Medical Center in California, USA. Presently, she is a cardiology fellow at the Southwest Healthcare in Southern California. She is a Research Scholar at the Lundquist Institute and a visiting researcher at the Scripps Clinic in La Jolla, CA, USA. Her research endeavours range from valvular heart disease to advanced cardiovascular imaging techniques, with a focus on cardiac CT and echocardiography.
