# Supplementary material for: Impact of technical, patient-related and measurement variables on serial Hounsfield unit–based quantitative coronary plaque analysis in computed tomography: time for a new chapter
Source: Eur Heart J Imaging Methods Pract. 2025 Jan 29;3(1):qyaf014. doi: 10.1093/ehjimp/qyaf014 (PMC11891479; doi:10.1093/ehjimp/qyaf014)
Supplement: qyaf014_Supplementary_Data [file qyaf014_supplementary_data.zip › Appendix figures and table.docx]

**Appendix Figures**

**Figure 1a**. Phantom coronary artery calcium volume and density with comparison with the ground truth, adaptive volume score (GE Smart Score 4.0) versus Callister volume score (Vital Images).


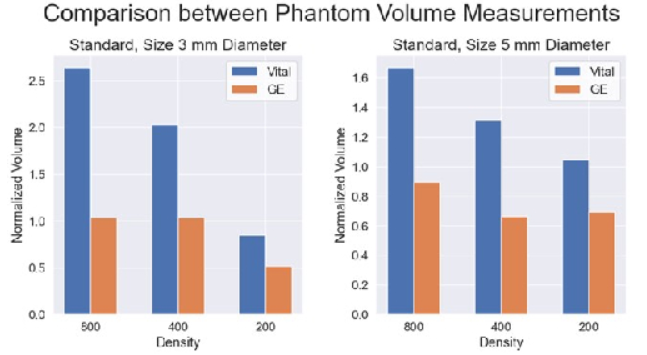


**Figure 2a.** Percent difference in the adaptive volume score (AVS) and conventional volume score (CVS) in the calcium volume versus Criqui plaque density in patients.


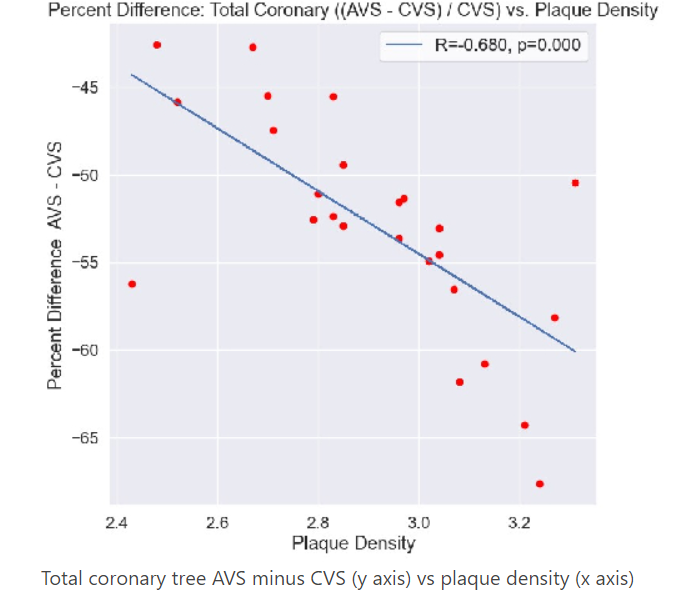


**Figure 3a**. Difference in LAP between HeartFlow (x axis) and AutoPlaque (y axis). Adapted

from Tzimas et al.^20^ .


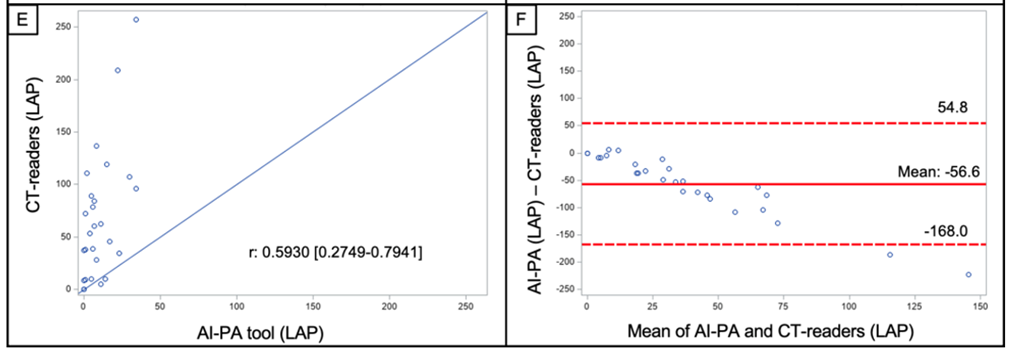


**Figure 4a.** Low energy sensitivity in adipose tissue and brain myelin in PCD-CT head image on silicon detector prototype.^88^


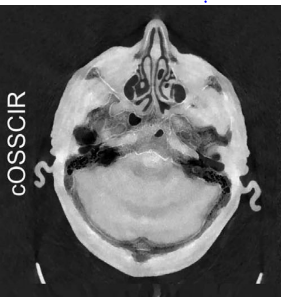


**Table 1a.** Difference in the total plaque, calcified plaque, non-calcified plaque, and low-attenuation plaque volume between HeartFlow and AutoPlaque. Adapted from Tzimas et

al.^20^ .

| **Variables** | **ICC (95% CI)** | ***P* value** |
| --- | --- | --- |
| **Total plaque** | **0.89 (0.7783–0.9471)** | **<0.0001** |
| **Calcified plaque** | **0.95 (0.8959–0.9763)** | **<0.0001** |
| **Noncalcified plaque** | **0.84 (0.6860–0.9220)** | **<0.0001** |
| **Low-CT-attenuation plaque** | **−0.14 (−0.4790 to 0.2354)** | **0.7617** |
