# Supplementary material for: Impact of technical, patient-related and measurement variables on serial Hounsfield unit–based quantitative coronary plaque analysis in computed tomography: time for a new chapter
Source: Eur Heart J Imaging Methods Pract. 2025 Jan 29;3(1):qyaf014. doi: 10.1093/ehjimp/qyaf014 (PMC11891479; doi:10.1093/ehjimp/qyaf014)
Supplement: qyaf014_Supplementary_Data [file qyaf014_supplementary_data.zip › Appendix text.docx]

**Appendix**

**Section 1**

**History of non-contrast computed tomography coronary calcium standardization and metrics and universally accepted phantom: review of the literature**

Since its introduction in 1990 with electron-beam computed tomography (EBCT), the non-contrast computed tomography (CT) coronary artery calcium (CAC) protocol has maintained absolute fidelity to the original scan technique proposed by Agatston and Janowitz^1^. However, the same cannot be said for coronary computed tomography angiography (CCTA) scanning and coronary plaque analysis. The success of coronary CT calcium in stratifying risk for major adverse cardiovascular events is, in large, part credited to the use of the same standardized technique published in the 1990 document: 130 kVp, 2.5–3-mm slice thickness, 25–35-cm field of view and Agatston–Janowitz scoring system. The simple fact that EBCT offered only one tube voltage dramatically simplified standardization.

The Multi-Ethnic Study of Atherosclerosis (MESA) has published well over 1,000 articles validating the value of coronary calcium in risk stratification and outcomes for major adverse cardiovascular events. From the beginning, these studies employed the use of external reference standards, which quantified measured Hounsfield unit (HU) to external calcium hydroxyapatite reference materials in different concentrations^2^. Nelson et al.^3^ found attenuation values to change scanner–vendor and subject body size, producing calcium score changes that are not due to true calcium burden differences. Phantoms allowed valid comparison between scans from different sites and scanners. Most recently, a MESA phantom-based vendor-neutral calibration tool for the Agatston score derived from seven different multi-slice CT systems and one electron-beam tomography system led to reclassification into higher risk categories with higher event rates in 11% of patients^4^.

Standardization of an additional calcium measurement was supplied by the interpolated Callister volume method in 1998^5,6^. A 2007 consortium of all four CT scan vendors resulted in the most rigorously validated method (calcium mass) with ground-truth measurement^5,6^. Using a commercially available CT calcium phantom, accurate calibration of CAC mass measurements was achieved with scanner- and thorax-size-specific calibration for each vendor. Superb agreement in the measurement of calcium mass in the phantom was met between all four vendors. The excellent reproducibility of calcium mass scores was confirmed in another study in 2010 comparing two identical CT scanners^7^. In 2021, van Praagh et al. published an open-source fully automated quantification method for coronary calcium on an anthropomorphic phantom validated on all four vendors of CT scanners and their proprietary analysis software^8^.

Despite the rigorous scientific validation, calcium mass measurements have not been adopted into routine clinical reports. Why is this so? Extremely few articles on cardiovascular outcomes related to calcium mass have been published. What are the technical requirements to measure coronary calcium mass? These include a one-time phantom calibration and CT vendor-specific calibration software.

In regard to the original interpolated coronary volume methodology proposed by Callister et al.^6,9,10^ in 1998, it was first shown in 2003^6,9,10^ and then again in 2009^6,9,10^ that both intermediate-to-high-density calcium (400–800 mg of hydroxyapatite/cm^3^) and lower tube voltage significantly overestimated the calcium volume by as much as 400%–600% relative to ground-truth phantom measurements. The accuracy for the conventional volume score (CVS) significantly decreased with decreasing tube voltage, with the highest and lowest accuracies at 140 and 80 kVp, respectively. This makes logical sense because higher tube energies result in less calcium blooming and less overestimation of the calcium volume^11^. Another study by Arnold et al. in 2010 found that the CVS overestimated the calcified plaque (CP) volume by as much as 580%, and they concluded that the observed heterogeneity of CP densities was due to energy-integrating detector CT (EID-CT) scanner measurement intrinsic errors inherent to the limited spatial resolution of these scanners as defined by the point spread function^12^. These studies demonstrate how calcium volume measurements have been known to be inaccurate relative to ground truth, often due to dense calcium and the use of lower tube voltages, which results in higher HU attenuation for hydroxyapatite and, therefore, blurring off the point spread function and overestimation of the plaque volume. Improved methods for calcium volume measurements to overcome blooming artefacts with ground-truth validation have also been reported. In 2009, Saur et al. studied a mesh-based volume measurement method using cardiac CT phantom with various cylindrical calcifications. They found that the mesh-based method was less dependent on tube voltages, reconstruction kernels and plaque density compared with the traditional CAC scoring method^10^. In 2023, Calicchio et al.^13^ conducted a pilot patient and phantom study (Figure 1a and 2a) in which a widely commercially available adaptive software (adaptive volume score [AVS]) was used for cardiac CT CAC volume quantification in mm^3^ that corrected for the significant overestimation related to the intermediate- and high-density calcium blooming artefact using the conventional interpolated full-width half-maximum technique traditional Callister volume method (CVS)^6^. Using the density measurement method of Criqui et al.^14^, the higher the calcium plaque density, the greater the difference between the AVS and CVS in patients because the AVS corrects for overestimation (Figure 2a).

**Section 2**

**A brief review of the spectrum of extensive variability in the reported absolute measurements of coronary tree coronary plaque focusing on LAP: biological versus technical?**

Identification of lipid-rich necrotic core (LAP) is central to identifying high-risk vulnerable plaques. We limited our brief review of LAP to publications that reported using the +30-HU LAP ceiling in cubic millimeters, unless otherwise noted. In multiple studies, a wide range of total coronary tree LAP volumetric values exist in the literature. Depending on the cardiovascular risk of the population and software analysis vendors, the total coronary tree LAP median values range from 0.0 ± 0.0 mm^315^ in 2,000 low-risk patients to 0.00 (high aortic lumen attenuation) and 0.39 (low aortic lumen attenuation) mm^3^ in 1,236 low- to intermediate-risk patients in the PARADIGM substudy^16^ and 39.0 mm^3^ in 40 post-acute coronary syndrome (ACS) patients^17^. For the non-HU approaches, a single individual plaque LAP volume as large as 258 mm^3^ has been reported in a control patient in the EVAPORATE trial^18^.

The 2020 SCCT consensus statement concluded that evidence from the literature is insufficient to establish the clinical use of serial quantitative plaque analysis^19^. For example, the PARADIGM serial plaque sub study was conducted in a low- to intermediate-risk group of 1,236 patients from multiple sites and different scanners and protocols^16^. The fixed HU threshold plaque analysis was employed: LAP, −30 to 30 HU; fibrofatty plaque (FFP), 31–130 HU; fibrous plaque, 131–350 HU; CP, >350 HU. Aortic lumen attenuation was grouped into three tertiles: low attenuation, <350 HU); intermediate attenuation, 350–500 HU; and high attenuation, >500 HU. At baseline, 70% of patients had a tube voltage of ≥120 kVp. Over 95% of patients with low lumen attenuation at baseline were scanned with ≥120 kVp; the majority of patients with high lumen attenuation at baseline were scanned with 100 kVp. On the second CT scan, 59% of patients were scanned with ≤100 kVp, resulting in a mean increase of 88 HU, with 49% of the patients with lumen attenuation in the third tertile at >500 HU. In contrast, only 19% had high lumen attenuation at baseline. At both baseline and follow-up, high lumen attenuation and the use of ≤100 kVp significantly decreased the total coronary tree LAP and FFP and increased the CP volume.

From this PARADIGM sub study, a significant difference in the total coronary tree LAP volume is observed as grouped by the lumen HU^16^. The median cubic millimeter volume of the total coronary tree LAP in all three attenuation tertiles was exceedingly low at baseline CCTA with wide interquartile ranges: 0.39 mm^3^ (4.61) in the lower tertile (<350 HU), 0.06 mm^3^ (1.96) in the intermediate tertile(350–500 HU) and 0.00 mm^3^ (0.28) in the higher tertile (>500 HU). Although the patients between the three tertiles may have different LAP volume, the statistically significant difference is clearly statistical noise and not biological. Perhaps even more importantly, these are truly tiny absolute volumes for the total coronary tree LAP, and the scan–rescan variability (not measured in this study) likely vastly exceeds these tiny values.

The same tube voltage was used in both scans in only 42% of the PARADIGM patients. A common denominator of studies exhibiting very low values (<1 mm^3^) of the total coronary tree LAP is the use of either lower tube voltages or higher lumen attenuation, often with fixed HU threshold software analysis^15,16,20^. In 448 patients with a variety of risks in six different fixed +30-HU LAP ceiling CCTA plaque analysis studies, the mean LAP was 0.6mm^3^ (median 0.0 mm^3^)^21,22,23,24,25,26^. Median aortic attenuation and tube voltage was 603 HU and 120 kVp, respectively. In the highest-risk population (ACS incident after CCTA, n = 21), the mean LAP was 1.8 mm^3^ (0.04% PAV based on the vessel volume denominator) versus control 0.9 mm^3^ (0.02% PAV)^21^. The same +30-HU LAP ceiling fixed HU software analyzed 303 CCTA patients before non-emergent ICA and FFR for a clinical indication^27^. The range of total coronary tree LAP varying by stenosis was 8.8–15.0 mm^3^, with a PAV of 0.20%–0.31% of the total vessel volume. Lumen attenuation was not reported.

The 30-HU threshold for LAP is recommended in the Coronary Artery Disease Reporting and Data System (CAD-RADS) version 2 expert consensus document and has been the most widely used across research studies and in clinical practice^28,29^. It was originally proposed in the intravascular ultrasound (IVUS)-validated publications by Motamoya et al. in 2007 who defined a +30-HU LAP ceiling at 135 kVp, at a coronary lumen contrast density of 258 ± 43 HU using a much slower injection and lower volume of less dense contrast compared with the current CCTA use^30,31^. The attenuation values for LAP, NCP and CP were 11 ± 12, 78 ± 21 and 516 ± 198 HU, respectively. More recent studies with higher iodine delivery rates have resulted in higher coronary lumen attenuation, for example, 367 HU^32^ at 120 kVp and 421 HU^33^ at 100–120 kVp. In the PARADIGM sub study by Takagi et al.^16^, over half of the patients had aortic lumen attenuation of >500 HU on the follow-up study.

In the Incident Coronary Events Identified by Computed Tomography (ICONIC) study, 234 patients underwent CCTA and were followed over time for the development of ACS and compared with controls using semi-automated plaque analysis software (QAngio CT; Medis, Leiden, The Netherlands). Statistically significant differences in plaque composition with increased prevalence values of the fibrofatty plaque (58.7 vs. 41.4 mm^3^, p = 0.009) and LAP (6.5 vs. 4.2 mm^3^, p = 0.026) volumes were detected in the group with ACS^34^. The percentage of LAP by the total vessel volume of the entire coronary tree was 0.3% (ACS) versus 0.2% (control) (p = 0.039). Lumen attenuation was not reported.

The EVAPORATE trial by Budoff et al. studied progression of the LAP volume over 18 months in 80 patients who received icosapent ethyl (IPE). The authors demonstrated significant changes between the study and placebo groups with regard to the log-transformed LAP volume, which decreased by 17% in the IPE subgroup (p = 0.0061), and fibrous and FFP volumes (p < 0.01). LAP was defined as −50 to + 50 HU^35^.

There is a lack of standardization in the calculation of plaque subtypes even within an individual plaque. Some reports and software vendors limit the individual plaque-reported LAP volume to the actual number of voxels that contain ≤30 HU. Therefore, if only a single voxel is <30 HU, then the cubic millimeter value of that single voxel will be reported as the amount of LAP in that individual plaque.

On the other hand, other software analyses have reported a different approach. Based on the histogram analysis, if ≥5.5% of voxels are <30 HU, then the entire plaque volume is labelled as LAP, even if an additional 94.5% of the voxels are >30 HU^36^. This cut point of 5.5% of pixels ≤ 30 HU allowed identification of lipid-rich plaques with a sensitivity of 95% and specificity of 80%. A more sophisticated 3D histogram analysis approach found the best cut point at ≤45 HU^37^.

As discussed and demonstrated most thoroughly by Takagi et al.^16^, a fixed HU threshold can be problematic. In fact, the use of fixed HU thresholds can have significant changes in the measured plaque volume with changes in the luminal iodine concentration and kilovoltage peak. These technique-induced HU changes result in technical, not biological, changes in plaque subtype measurements. Several authors have pointed out that luminal attenuation and tube voltage are ‘different sides of the same coin’ that have a deep impact on plaque characterisation^38^.

In 2010, Achenbach et al. showed that NCP is significantly affected by image reconstruction. Higher CT attenuation and, therefore, higher NCP volumes result from diminishing spatial resolution (larger voxels) with softer kernel or thicker slices^39^. The authors concluded: ‘Using absolute CT attenuation values for plaque characterization may therefore be problematic’. The same conclusions were made in ex vivo studies of coronary arteries to be reviewed more extensively in Part 2. Even the original seminal study by Motoyama et al.^30^ found that NCP on CT with a slice thickness of 0.5 mm had a lower density and CP had a higher density than the data from CT with a slice thickness of 1 mm.

One large PARADIGM sub study by Lee et al.^40^ has examined serial plaque progression at ≥2 years with the two scans using the same tube voltage. A total of 1,476 lesions were analyzed. Although the follow-up TP population statistics between scans showed statistically significant changes in the mean plaque volumes (except LAP), the scan–rescan variability has not yet been reported in any PARADIGM study. It remains unclear from this population data as to its applicability to serial scans in an individual patient. Would a 5-mm^3^ change in any compositional plaque volume in an individual patient be measurable as true biological change and not scan–rescan expected measurement uncertainty?

Reliable identification of any early coronary plaque is highly desirable. Distinguishing small plaques from artefacts related to background noise can be difficult even with excellent image quality. Another PARADIGM sub study used a different software analysis vendor (machine learning) rather than the semi-automated software used in all other PARADIGM sub studies^41^. They defined ‘small’ plaque (CP, NCP or LAP) as <50 mm^3^ and identified persistence at the same location in 87% of 437 plaques from 99 patients, proving that these small lesions were not artefact. A value of 50 mm^3^ is only 0.05 mL (0.05 cm^3^) of volume, equivalent to a single drop of water! All of the persistent small plaques were CP or NCP, and no measurable LAP was found. These are tremendously useful data with machine learning to identify true absence of no plaque versus small plaque because expert reader intra-observer and inter-observer agreement is suboptimal^42^. The PARADIGM registry represents a lower-risk population, and it should be expected that LAP quantification is lower than that in the higher-risk populations^43^. The population was primarily of Asian ethnicity (70.7% in the largest study of 1,602 patients), and the applicability of some findings to other ethnicities is unknown. All PARADIGM sub-studies reported very low total coronary tree LAP volumes, all under the individual plaque 2.3-mm^3^ lower limit for a fixed +30-HU accuracy to near-infrared spectroscopy–IVUS-established lipid-rich plaque by the INVICTUS trial^44^.

In DISCOVER VP^45^, eight post-mortem sudden coronary death hearts were evaluated with CCTA and histological analysis. LAP was defined as a pixel of <30, 45, 60, 75 or 90 HU cutoff (LAP floor minus 30 HU) within the 37 coronary plaques analyzed. Although the highest specificity for identifying lipid-rich plaque was shown with a cutoff of <30 HU (88.2%), both the receiver operating characteristic (ROC) area under the curve (AUC) (0.54) and cross-sectional area agreement with histology were best with <75 HU. In 55 LAPs from five ex vivo hearts, Schlett et al.^46^ found that the relative area of plaque of <60 HU from a quantitative histogram analysis had good accuracy versus histology to detect LAP independent of differences in luminal contrast enhancement

Marwan et al.^36^ attempted to improve the LAP diagnostic accuracy by counting the number of pixels corresponding to the plaque types on histograms of HU. If >5.5% of voxels were <30 HU, the ROC curve analysis identified LAP with a sensitivity of 95% (38/40; 95% CI, 83–99) and specificity of 80% (12/15; 95% CI, 52–96) (AUC, 0.9; 95% CI, 0.7–1.0). The negative and positive predictive values were 86% (12/14; 95% CI, 57–98) and 93% (38/41; 95% CI, 80–98), respectively.

**Section 3**

**Scan-specific method of Dey et al**^47^

In the SCOT-HEART post hoc analysis^48^, the TPV, CP and NCP were measured with the this scan-specific adaptive technique adjusting to local attenuation. As noted in the methods, where they cite their previous work^37^, LAP was defined by three-dimensional (3D) attenuation histogram-based quantification with a fixed cutoff of +30 HU as opposed to an individual voxel or mean HU analysis cutoff of +30 HU. Histogram-based LAP analyses are independent of cross-sectional area regions of interest. LAP was measured with a different ***adaptive*** technique (3D histogram analysis) compared with the typical ***fixed*** mean HU or individual voxel HU-based techniques used by other vendors. Plaque burden (as a percentage) was calculated for each of the total plaque (TP), NCP, LAP and CP, by dividing the relevant plaque volume by the vessel volume *of the region assessed, multiplying by 100 and summing on a per-patient basis.* This definition of the denominator for the percent atheroma volume is **very** different from the total coronary tree vessel volume used by several other vendors. These unique analytic techniques explain why the statistically significant results of this study (7.5% LAP burden in the myocardial infarction [MI] group vs. 4.1% in those without MI) cannot be compared with those of other vendors.

**Section 4**

**Description of adaptive mode of deGraaf et al.**^49^

From the description of the methods in the ARCHITECT trial^50^:

“A linear trend line is fitted through the mean lumen intensity. Next, the threshold for LAP is defined as 200 HU below this estimate with a maximum of 75 HU, and the dense calcium threshold is defined as 100 HU above this estimate with a maximum of 450 HU. The cutoff for LAP is set at 200 HU below this estimate up to 75 HU, and the dense calcium cutoff is set at 100 HU above this estimate up to 450 HU. The threshold between fibrotic tissue and fibro-fatty tissue is set on 20% of the difference between the LAP and dense calcium threshold. Additionally, because the lumen intensity is lower in parts of a severe stenosis, the LAP cutoff value is locally decreased with 125% of the difference between the estimate and real lumen intensities. In contrast, the lumen intensity is higher in calcified parts due to blooming artefacts. Therefore, the dense calcium cutoff value is locally increased with 25% of the difference between the estimate and real lumen intensities”.

**Section 5**

**Intervendor comparisons**

Lin et al.^51^ examined plaque analysis agreement between a newer deep learning version and the original semi-automated software with scan-specific settings^47^. In 1,901 coronary lesions, the results obtained by machine learning demonstrated a close correlation with the results obtained by expert readers using semi-automated software for the measured TPV (intraclass correlation coefficient [ICC], 0.964; 95% confidence interval [CI], 0.960–0.967), CP (ICC, 0.945; 95% CI, 0.939–0.950) and NCP (ICC, 0.938; 95% CI, 0.932–0.944; all p < 0.0001). A good correlation was also noted in the results for the LAP volume (ICC, 0.810; 95% CI, 0.786–0.831). The mean difference in the TPV on the BA analysis between deep learning and semi-automated software experts was 5.41 mm^3^ (95% limits of agreement, −114.74 to 125.56 mm^3^). For NCP and CP, the mean differences were 2.98 and 2.43 mm^3^, with 95% limits of −114.51 to 120.42 and −43.57 to 48.46 mm^3^, respectively. The durations of analysis time were 5.65 s for deep learning using a graphics processing unit and 3.82 min using a central processing unit. Experts using the semi-automated software took 25.7 min.

Tzimas et al.^20^ investigated the intervendor differences between an AI-based software HeartFlow AI-PA tool (HeartFlow, Mountain View, CA, USA) and Autoplaque. The total plaque, CP, NCP and LAP volume were compared in 27 patients. The results for all plaque compositions are shown in Table 1 and Figure 3a. Both vendors used a fixed ceiling threshold of +30 HU for LAP. In the limited sample analyzed (n = 27), low-CT-attenuation plaque had an ICC of −0.14 (95% CI, −0.4790 to 0.2354; p = 0.7617). A BA bias difference of 56.6 mm^3^ in LAP was noted between the two vendor’s software, with 95% confidence limits of −168 to 54.8 mm^3^. This LAP difference may indicate the absence of a standardized measurement between these two vendors. This discrepancy could be further investigated by phantom validation and calibration studies.

An even more revealing comparison of vendors is found in two different analyses of the EVAPORATE trial, 2020 (QAngio CT)^35^ and 2023 (Elucid)^18^. The QAngio CT software was employed for LAP with fixed +30-HU mode, and Elucid used machine learning validated by histology without relying on quantification of HU^52,53^. With QAngio CT, the baseline placebo group total tree LAP was 6.31 mm^3^, and with Elucid, the LRNC was 83 mm^3^. The authors attribute this difference to the objective definition of the LRNC based on histology, as opposed to the variable definitions and measurements of LAP^18^.

Mancini et al ^54^ analyzed the correlations between two different plaque measurement methods in 24 patients: QAngio CT (fixed and adaptive) versus Vital (fixed). Calcified plaque was highly consistent when comparing adaptive and fixed threshold techniques. However, the correlations became progressively weaker when examining NCP and particularly LAP, with the latter showing either statistically insignificant or only marginally significant correlations. The LAP comparison of both vendors at +30 HU was particularly striking. They concluded QAngio CT mixed plaque and LAP volumes appear to be systematically underestimated, compared to Vital. Of course, in the absence of a reference standard, one could also conclude Vital overestimated plaque volumes relative to QAngio CT. The bias becomes more pronounced as the volumes of mixed plaque and LAP increase, highlighting a significant limitation in the comparability of these measurements across these two different platforms.

**Section 6**

**Scan-Rescan analyses**

Scan–rescan reliability measurements provide clinicians with confidence that the measurement metric from the CT scanner results in the same outcome each time for an individual patient^55^. Both the Bland–Altman analyses^56^ and repeatability coefficients (RC), also known as the smallest real difference^57^ (threshold), are considered appropriate methods for calculating test–retest reliability and are directly related to one another^58^. The Bland–Altman analysis shows the difference, systematic bias and 95% limits of agreement between the two measurements in the test’s units of measurement (e.g. cubic millimeter of plaque). The RC is simply the value below which the absolute differences between two measurements would lie with 0.95 probability. Stated differently, a measured change needs to be above this value to be ‘real’. It is calculated by simply multiplying the within-subject standard deviation of the repeated measurements times two. This represents the smallest possible change in a metric that represents true change. Indeed, this is extremely relevant to serial coronary plaque CT research. We consider measurement of this metric (CR) a requirement for proving biological change.

**Quantitative plaque analysis scan–rescan studies with the same kilovoltage peak**

Cheng et al. (2009) studied 30 patients 124 days apart with quantitative plaque analysis and manual tracings. They found a wide range of the 95% Bland–Altman confidence limits for noncalcified plaque (NCP) when expressed as a percent of the mean volume of the two scans. For one reader, this scan–rescan percent variability ranged from 164% to 216%, and for the other, this ranged from 154% to 210%^59^. These differences in agreement are large compared with the mean compositional plaque volume. The total plaque volumes (TPVs) in 77% of segments were small (i.e. <50 mm^3^). This results in small absolute variations becoming relatively large.

In a study of 20 patients who underwent two CCTA examinations within 100 days, Schuhbeck et al.^60^ found no significant differences in the plaque volumes and very good interscan correlation of these volumes (Pearson's correlation coefficients, r = 0.92, r = 0.90 and r = 0.96 for the TPV and NCP and CP volumes, respectively).

Ovrehus et al.^61^ also found excellent interscan, interobserver and intra-observer agreements for the NCP and CP volumes. The interscan mean percentage differences in the NCP and CP volumes were 0.1% (*p* = 0.8) and 1.9% (*p* = 0.19), with limits of agreement of ±11% and ±48.5%^61^. Low-attenuation plaque (LAP) was not reported.

In 2016, Symons et al.^62^ compared the scan–rescan variability of plaque volume measurements a mean of 19 days apart in 20 patients for same-vendor scan–rescan variability and 20 additional patients for different-vendor scan–rescan variability. The intraclass correlation coefficients (ICCs) were excellent for the same vendors—TPV and NCP and CP volumes (ICCs, 0.97, 0.97 and 0.95, respectively)—but lower for different vendors (0.77, 0.94 and 0.61, respectively). LAP was not reported. They added: ‘Considering a clinical trial intended to show a change of 5% in noncalcified plaque volume over time with a power of 90%, 217 patients would be needed for lesion-based analysis of noncalcified plaque for follow-up with the same scanner’.

Lee et al.^63^ reported scan–rescan agreement in all compositional plaque volumes for 95 patients (847 pairs of segments) with the same tube voltages. The Bland–Altman analysis demonstrated a mean difference in the TPV per-segment of 1.1 ± 17.7 mm^3^ (95% confidence interval [CI], −2.5 to 2.6 mm^3^).

Meah et al.^64^ found excellent agreement for TPV and CP and NCP volume measurements in advanced CAD using a semi-automated method. Twenty patients with known multivessel coronary disease (95% with previous coronary revascularization) underwent serial EID-CT scan 2 weeks apart using identical reconstruction protocols. The TPV was very large—2,063 ± 1,246 mm^3^ on scan one and 2,027 ± 1,223 mm^3^ on scan two. The mean LAP volumes were 13 ± 13 and 10 ± 13 mm^3^ on the first and second scans, respectively, and the mean NCP volumes were 1,765 ± 910 and 1,760 ± 1,928 mm^3^, respectively. The scan–rescan Bland–Altman bias for LAP was −2.6 mm^3^, with 95% confidence limits from −16 to +11 mm^3^. The scan–rescan Bland–Altman bias for NCP was −4.3 mm^3^, with 95% confidence limits from −418 to +409 mm^3^. The CRs were 13.1 and 413.1 mm^3^, respectively. They concluded that NCP was a more reliable plaque metric to measure for clinical trials than LAP because the wide relative limits of agreements, despite the narrow absolute limits of agreements noted with LAP, reflect the much smaller volumes measured.

Using an AI-enabled plaque analysis software, Tomasino et al ^65^ found excellent per-patient agreement of TPV, CP, NCP, and LAP in 39 high-risk patients between systole and diastole within the same cardiac cycle. The mean TPV was 978 mm^3^. Lin’s and repeatability coefficients (RC)^55^ were 0.97, 0.99, 0.96, and 0.98, and 121.8, 33.4, 118.4, and 57.6 mm^3^, respectively. Translating these results, if one of these high-risk patients were followed for NCP serial change with different phases of the cardiac cycle, the difference would need to exceed 118 mm^3^ to be considered biological.

Obuchowski and Buckler^66^ performed simulation studies to demonstrate methods to produce unbiased estimators of repeatability and valid statistical tests without performing test–retest studies. They did not have actual test–retest data with a reference standard to validate this simulation. Their simulations covered a wide range of conditions to include the variability of their reference standard itself (carotid endarterectomy plaque histology). The imprecision in the reference standard itself is due to decalcification of the specimen with formalin fixation and location misalignment of the histological specimen with the location of the measurement. The endarterectomy specimen does not consist of the entire vessel wall but only the excised plaque^67^. The proximal internal carotid artery diameter is larger than the proximal coronary artery diameters. Their proposed repeatability estimates enable determining the minimum change over time that likely reflects an actual change rather than mere measurement error. Specifically, the minimum meaningful changes with the Elucid analysis were the following: CALC area, changes of >2.1 mm^2^; lipid-rich necrotic core area, changes of >5.2 mm^2^; and IPH area, changes of > 4.2 mm^2^.

**Quantitative plaque analysis scan–rescan studies with different kilovoltage peaks**

Lee et al.^63^ reported scan–rescan analysis in all compositional plaque volumes and TPVs in 24 patients, with a median difference of 20 kVp in tube voltage between scans. The mean difference in the TPV per-segment was 0.4 ± 3.4 mm^3^, with a Bland–Altman 95% CI of −7.1 to +6.2 mm^3^. This CI was compared with that of a different cohort of 95 patients with the same tube voltage: mean difference in the TPV per-segment of 1.1 ±17.7 mm^3^ (95% CI, −2.5 to 2.6 mm^3^). Obviously, this supports the belief that maintaining the same tube voltage on serial scans results in better precision of per-segment plaque measurement. The mean TPV of the two scans was 62.1 mm^3^. With different tube voltages, the TPV, CP, fibrous, fibrofatty, and LAP means (± standard deviations [SDs]) of differences between the two scans were 0.4 ± 3.4, 0.0 ± 4.6, 0.6 ± 4.6, 0.1 ± 1.6 and 0.2 ± 1.9, respectively. The TPV coefficient of variation (COV) was 16% (the ratio of the SD of the differences divided by the mean of the two measurements). As the compositional mean plaque volumes became smaller, the COVs increased. The COVs for the CP, fibrous, fibrofatty and LAP volumes were 20.8%, 63%, 138% and 560%, respectively. This reinforces the conclusions of Meah et al.^64^.

**Section 7**

**Realistic expectations for solutions** **in serial plaque CCTA measurements with spectral EID-CT and PCD-CT**

Many, but not all, problems currently reviewed in this document with polychromatic EID-CT will be improved with PCD-CT. To date, over 100 clinical sites are installed worldwide. Unlike EID-CT, where only photon energies above 30 keV are processed, PCD-CT counts all X-ray quanta with equal weight, including lower energies^68^. This leads to higher tissue contrast in scans with iodinated contrast media^68^. With the virtual elimination of electronic noise by the removal of scintillating light photons in the imaging chain, all areas of CT performance currently hindering plaque quantification will be improved and reviewed in detail in Part 2 of this review. This optimism is based on multiple coronary plaque phantom^69–73^ and ex-vivo studies^74^. More recently, Koons et al. found that ultrahigh-resolution PCD-CT decreased CCTA percentage diameter stenosis by 11% compared with EID-CT, resulting in 13 of 34 stenoses being downgraded in the stenosis severity category^75^. Another work by Koons et al^76^ developed a super resolution convolutional neural network (CNN) for EID-CT: ILUMENATE (Improved LUMEN visualization through Artificial super-resoluTion imagEs), creating a high resolution (HR) image from EID-CT simulating UHR PCD-CT. ILUMENATE improved the quality of EID-CT images bringing the spatial resolution closer to UHR PCD-CT images by reducing the calcium blooming. Halfmann et al. found that PCD-CT with ultrahigh resolution (0.2 mm) led to a lower percentage diameter stenosis from calcific plaque than PCD-CT with standard resolution (0.6 mm) and clinically relevant rates of reclassification^77^. As summarized by McCollough et al.^78^ and Danielsson et al.^79^, PCD-CT several major advantages, including the following.

Improvement in the limited spatial resolution at 125–150 µm allows submillimeter delineation of structures and improves geometric quantification, with diminished partial volume averaging, thus resulting in potential for improved measurement of the coronary plaque volumes. Acquisition is intrinsically multi-energy acquisitions, at all times, with superior spectral sensitivity compared with EID-CT dual energy. This allows the following:

a) VMIs at photon energy levels from 40 to 180 keV. The CT numbers for a given material (e.g. iodine) at a specific VMI energy (e.g. 50 keV) should be stable and exact across different vendors, models, beam filtrations and tube voltages. This requires verification by scanning multienergy phantoms in cardiac acquisition modes. Phantoms can also measure CT number stability which is required for good quantitative CT, including serial CCTA plaque analysis^68^. The direct conversion of photon energies into electrical signals may allow improved/routine material decomposition capabilities. This needs to be proven by phantoms. Combined with the rapidly developing deep learning and artificial intelligence applications, CT will convert to a truly quantitative modality^80^. Deep silicon PCD-CT showed lower CT number variability as a function of body size than polychromatic and dual-energy EID-CT^81^. The CT numbers with PCD-CT were closer to the ideal CT number calculated from the National Institute of Standards and Technology(NIST) photon cross-sectional database^82^. These authors showed the crucial impact of body size on CT number stability for all three technologies. For all objects within the phantom, the CT number increased with decreasing thorax diameter, an observation also found by Takagi et al. in the PARADIGM CCTA sub study, where a lower body mass index was associated with higher iodine lumen attenuation^16^. Why is photon counting more accurate than conventional CT at measuring the CT number? This is because direct measurement of individual photon energies with PCD-CT results in more accurately determining the effective beam energy. In EID-CT, it is not possible to directly measure the effective energy of the X-ray beam that reaches the detector. Instead, the different EID-CT vendors use ‘one body size-fits-all’ proprietary beam hardening corrections^81^ to estimate the CT number. This is a true fundamental weakness of EID-CT and leads to intervendor variability of CT number measurements for the same object in the same phantom^83^.

b) Atomic number maps and histogram analyses^84,85^

provide a new visualization and characterization tool for atherosclerotic plaque.

c) Material-specific density maps, for example, iodine, calcium and water, and subtracted material maps, such as non-calcium^68^ and non-iodine (black-blood CTA)^68^.

d) K-edge contrast agents, for example, simultaneous CCTA imaging of iodine (for coronary arteries) and late gadolinium enhancement of the myocardial scar (pre-scan injected 5 min earlier). It has even been predicted that PCD-CT will replace late gadolinium enhancement cardiac MRI for the detection of myocardial scar^86^.

Yet, another advantage of PCD-CT is that EID-CT scanners typically use an energy threshold to discriminate between the X-ray photons of interest and noise. The threshold is set to a specific energy level, typically approximately 30 keV, above which photons are considered part of the useful signal for image formation, whereas photons below this threshold are often treated as noise and not used in creating the final CT image. This thresholding in EID-CT helps improve the image quality and reduces unwanted noise. With PCD-CT, the traditional EID-CT energy threshold (<30 keV) is eliminated. PCDs can distinguish between different energy levels of X-ray photons individually, allowing for more precise control over the detected signals. This eliminates the need for a fixed energy threshold and provides better flexibility in acquiring and using data, improving image quality and diagnostic capabilities. This allows the user to emphasize the low-energy photons, which may improve the contrast-to-noise ratio^87^. An example of this low energy sensitivity in adipose tissue in a head CT image on a silicon detector-based prototype photon counting was published by Schmidt et al.^88^(Figure 4a). Low-energy photons of <30 keV will now be participating in soft tissue contrast formation, creating a ‘new imaging library’ for our brain^89^.

‘Photon-counting CT currently produces an approximate doubling of spatial resolution (around 0.25-mm resolution). This is essential for accurate and reproducible analysis of plaque volume and lumen analysis for small coronary vessels less than 2 mm in diameter’. CAD-RADS version 2.0 requires all vessels with a diameter of >1.5 mm to be graded for stenosis severity. More accurate CT numbers in each voxel will be produced for more accurate quantitative analysis. The blooming effects of CP will be reduced by the superior spatial resolution^69,70^, and spectral energy binning can produce lumen free of iodinated contrast medium. The CAD-RADS groups of stenosis descriptions (i.e. 25%–49%) can be narrowed with the doubling of voxel spatial resolution^86^.

**References**

1. Agatston AS, Janowitz WR, Hildner FJ, Zusmer NR, Viamonte M Jr, Detrano R. Quantification of coronary artery calcium using ultrafast computed tomography. *J Am Coll Cardiol* 1990;**15**:827–832.

2. Carr JJ, Nelson JC, Wong ND, McNitt-Gray M, Arad Y, Jacobs DR Jr, Sidney S, Bild DE, Williams OD, Detrano RC. Calcified coronary artery plaque measurement with cardiac CT in population-based studies: standardized protocol of Multi-Ethnic Study of Atherosclerosis (MESA) and Coronary Artery Risk Development in Young Adults (CARDIA) study. *Radiology* 2005;**234**:35–43.

3. Nelson JC, Kronmal RA, Carr JJ, McNitt-Gray MF, Wong ND, Loria CM, Goldin JG, Williams OD, Detrano R. Measuring coronary calcium on CT images adjusted for attenuation differences. *Radiology* 2005;**235**:403–414.

4. Werf NR van der, Dobrolinska MM, Greuter MJW, Willemink MJ, Fleischmann D, Bos D, Slart RHJA, Budoff M, Leiner T. Vendor Independent Coronary Calcium Scoring Improves Individual Risk Assessment: MESA (Multi-Ethnic Study of Atherosclerosis). *JACC Cardiovasc Imaging* 2023;**16**:1552–1564.

5. McCollough CH, Ulzheimer S, Halliburton SS, Shanneik K, White RD, Kalender WA. Coronary artery calcium: a multi-institutional, multimanufacturer international standard for quantification at cardiac CT. *Radiology* 2007;**243**:527–538.

6. Callister TQ, Cooil B, Raya SP, Lippolis NJ, Russo DJ, Raggi P. Coronary artery disease: improved reproducibility of calcium scoring with an electron-beam CT volumetric method. *Radiology* 1998;**208**:807–814.

7. Dijkstra H, Greuter MJW, Groen JM, Vliegenthart-Proença R, Renema KWK, Lange F de, Oudkerk M. Coronary calcium mass scores measured by identical 64-slice MDCT scanners are comparable: a cardiac phantom study. *Int J Cardiovasc Imaging* 2010;**26**:89–98.

8. Praagh GD van, Werf NR van der, Wang J, Ommen F van, Poelhekken K, Slart RHJA, Fleischmann D, Greuter MJW, Leiner T, Willemink MJ. Fully automated quantification method (FQM) of coronary calcium in an anthropomorphic phantom. *Med Phys* 2021;**48**:3730–3740.

9. Hong C, Bae KT, Pilgram TK. Coronary artery calcium: accuracy and reproducibility of measurements with multi-detector row CT--assessment of effects of different thresholds and quantification methods. *Radiology* 2003;**227**:795–801.

10. Saur SC, Alkadhi H, Desbiolles L, Székely G, Cattin PC. ACCURATUM: improved calcium volume scoring using a mesh-based algorithm--a phantom study. *Eur Radiol* 2009;**19**:591–598.

11. Van Hedent S, Große Hokamp N, Kessner R, Gilkeson R, Ros PR, Gupta A. Effect of Virtual Monoenergetic Images From Spectral Detector Computed Tomography on Coronary Calcium Blooming. *J Comput Assist Tomogr* 2018;**42**:912–918.

12. Arnold BA, Budoff MJ, Child J, Xiang P, Mao SS. Coronary calcium test phantom containing true CaHA microspheres for evaluation of advanced CT calcium scoring methods. *J Cardiovasc Comput Tomogr* 2010;**4**:322–329.

13. Rosendael Samantha R. Spierling Bagsic Sanjeev Bhavnani Austin A. Robinson Elizabeth Epstein Bradley Patay Eric Gros Jorge Gonzalez George Wesbey FCSNEHA van. Groundtruth accuracy of an adaptive HU commercial coronary calcium volume measure vs conventional commercial fixed HU calcium volume measure in 120 kVp 2.5 mm thick cardiac CT slices: a pilot phantom and patient study. *ESCR-ESTI Joint Meeting 2023*. https://escr-esti2023.smart-abstract.com/eposter/#/posters/84 (January 22, 2024)

14. Criqui MH, Denenberg JO, Ix JH, McClelland RL, Wassel CL, Rifkin DE, Carr JJ, Budoff MJ, Allison MA. Calcium density of coronary artery plaque and risk of incident cardiovascular events. *JAMA* 2014;**311**:271–278.

15. Nasir K, Lahan S, Maqsood M, Budoff M, Earls J, Min J, Feldman D, Feldman T, Cury R, Blaha M, Blankstein R, Shaw L, Shah S, Cainzos M, Fialkow J. Variation Of Coronary Atherosclerosis Across Spectrum Of Increasing Cac Scores Using Quantitative Ct Plaque Analysis: Miami Heart Study (miheart) At Baptist Health South Florida. *J Cardiovasc Comput Tomogr* 2023;**17**:S41–S42.

16. Takagi H, Leipsic JA, Indraratna P, Gulsin G, Khasanova E, Tzimas G, Lin FY, Shaw LJ, Lee S-E, Andreini D, Al-Mallah MH, Budoff MJ, Cademartiri F, Chinnaiyan K, Choi JH, Conte E, Marques H, Araújo Gonçalves P de, Gottlieb I, Hadamitzky M, Maffei E, Pontone G, Shin S, Kim Y-J, Lee BK, Chun EJ, Sung JM, Virmani R, Samady H, Stone PH, Berman DS, Narula J, Bax JJ, Chang H-J. Association of Tube Voltage With Plaque Composition on Coronary CT Angiography: Results From PARADIGM Registry. *JACC Cardiovasc Imaging* 2021;**14**:2429–2440.

17. Vaidya K, Arnott C, Martínez GJ, Ng B, McCormack S, Sullivan DR, Celermajer DS, Patel S. Colchicine therapy and plaque stabilization in patients with acute coronary syndrome: A CT coronary angiography study. *JACC Cardiovasc Imaging* 2018;**11**:305–316.

18. Buckler AJ, Doros G, Kinninger A, Lakshmanan S, Le VT, Libby P, May HT, Muhlestein JB, Nelson JR, Nicolaou A, Roy SK, Shaikh K, Shekar C, Tayek JA, Zheng L, Bhatt DL, Budoff MJ. Quantitative imaging biomarkers of coronary plaque morphology: insights from EVAPORATE. *Front Cardiovasc Med* 2023;**10**:1204071.

19. Shaw LJ, Blankstein R, Bax JJ, Ferencik M, Bittencourt MS, Min JK, Berman DS, Leipsic J, Villines TC, Dey D, Al’Aref S, Williams MC, Lin F, Baskaran L, Litt H, Litmanovich D, Cury R, Gianni U, Hoogen I van den, R van Rosendael A, Budoff M, Chang H-J, E Hecht H, Feuchtner G, Ahmadi A, Ghoshajra BB, Newby D, Chandrashekhar YS, Narula J. Society of Cardiovascular Computed Tomography / North American Society of Cardiovascular Imaging - Expert Consensus Document on Coronary CT Imaging of Atherosclerotic Plaque. *J Cardiovasc Comput Tomogr* 2021;**15**:93–109.

20. Tzimas G, Gulsin GS, Everett RJ, Akodad M, Meier D, Sewnarain K, Ally Z, Alnamasy R, Ng N, Mullen S, Rotzinger D, Sathananthan J, Sellers SL, Blanke P, Leipsic JA. Age- and Sex-Specific Nomographic CT Quantitative Plaque Data From a Large International Cohort. *JACC Cardiovasc Imaging* 2023.

21. Rodriguez Ruiz C, Hermel M, Miller G, Chang CW, Salerno M, Van Rosendael A, Epstein E, Joye C, Spierling Bagsic SR, Newlander S, Bhavnani S, Robinson A, Gonzalez J, Wesbey GE. Are artificial intelligence derived coronary atherosclerotic characteristics associated with subsequent development of acute coronary syndrome (ACS). *Eur Heart J Cardiovasc Imaging* 2023;**24**:jead119.164.

22. Rodriguez Ruiz C, Spierling Bagsic S, Rosendael A van, Miller G, Salerno M, Hermel M, Newlander S, Bhavnani S, Robinson A, Gonzalez J, Wesbey G. No Particular Association Found Between LDL-p By NMR And Coronary Plaque Characteristics When LDL Is Normal. *J Cardiovasc Comput Tomogr* 2023;**17**:S9.

23. Hermel M, Rodriguez Ruiz C, Rosendael A van, Miller G, Bagsic S, Patay B, Hu E, Epstein E, Joye C, Newlander S, Salerno M, Bhavnani S, Gonzalez J, Robinson A, Wesbey G. Are Artificial Intelligence Derived Coronary Atherosclerotic Characteristics Associated With The Future Development Of Atrial Fibrillation. *J Cardiovasc Comput Tomogr* 2023;**17**:S41.

24. Chiou A, Hermel M, Miller G, Bagsic S, Rosendael A van, Udoh E, Sidhu R, Kosturakis R, Aziz M, Ruiz CR, Khadivi B, Newlander S, Brown JP, Charlat ML, Teirstein PS, Stinis C, Schatz RA, Price MJ, Cavendish JJ, Salerno M, Robinson A, Bhavnani SP, Patay B, Gonzalez JA, Wesbey GE. Cleerly^TM^ vs. Heartflow ^TM^ vs. Site read in the per-vessel prediction of adenosine ffr≤0.80 with plaque features associated with false positives. *J Am Coll Cardiol* 2023;**81**:1384.

25. Calicchio F, Hu E, Newlander S, Van Rosendael A, Epstein E, Robinson A, Bhavnani S, Patay B, Gonzalez J, Wesbey G. How Much Does Tube Voltage Affect Plaque Volume And Composition? 140 Vs 100 Kvp Four Seconds Later. *J Cardiovasc Comput Tomogr* 2024;**18**:S24.

26. Mansour C, Pelter M, Hu E, Chang C, Rosendael A, Hermel M, Epstein E, Bagsic S, Bhavnani S, Robinson A, Newlander S, Patay B, Nayak K, Gonzalez J, Wesbey G. AI Evaluation Of Stenosis On Coronary CTA: Real World Comparison With Quantitative Coronary Angiography. *J Cardiovasc Comput Tomogr* 2024;**18**:S7.

27. Min JK, Chang H-J, Andreini D, Pontone G, Guglielmo M, Bax JJ, Knaapen P, Raman SV, Chazal RA, Freeman AM, Crabtree T, Earls JP. Coronary CTA plaque volume severity stages according to invasive coronary angiography and FFR. *J Cardiovasc Comput Tomogr* 2022;**16**:415–422.

28. Matsumoto H, Watanabe S, Kyo E, Tsuji T, Ando Y, Eisenberg E, Otaki Y, Manabe O, Cadet S, Slomka PJ, Tamarappoo BK, Berman DS, Dey D. Improved Evaluation of Lipid-Rich Plaque at Coronary CT Angiography: Head-to-Head Comparison with Intravascular US. *Radiol Cardiothorac Imaging* 2019;**1**:e190069.

29. Cury RC, Leipsic J, Abbara S, Achenbach S, Berman D, Bittencourt M, Budoff M, Chinnaiyan K, Choi AD, Ghoshhajra B, Jacobs J, Koweek L, Lesser J, Maroules C, Rubin GD, Rybicki FJ, Shaw LJ, Williams MC, Williamson E, White CS, Villines TC, Blankstein R. CAD-RADS^TM^ 2.0 - 2022 Coronary Artery Disease - Reporting and Data System An Expert Consensus Document of the Society of Cardiovascular Computed Tomography (SCCT), the American College of Cardiology (ACC), the American College of Radiology (ACR) and the North America Society of Cardiovascular Imaging (NASCI). *Radiol Cardiothorac Imaging* 2022;**4**:e220183.

30. Motoyama S, Kondo T, Anno H, Sugiura A, Ito Y, Mori K, Ishii J, Sato T, Inoue K, Sarai M, Hishida H, Narula J. Atherosclerotic plaque characterization by 0.5-mm-slice multislice computed tomographic imaging. *Circ J* 2007;**71**:363–366.

31. Motoyama S, Kondo T, Sarai M, Sugiura A, Harigaya H, Sato T, Inoue K, Okumura M, Ishii J, Anno H, Virmani R, Ozaki Y, Hishida H, Narula J. Multislice computed tomographic characteristics of coronary lesions in acute coronary syndromes. *J Am Coll Cardiol* 2007;**50**:319–326.

32. Maffei E, Martini C, Arcadi T, Clemente A, Seitun S, Zuccarelli A, Torri T, Mollet NR, Rossi A, Catalano O, Messalli G, Cademartiri F. Plaque imaging with CT coronary angiography: Effect of intra-vascular attenuation on plaque type classification. *World J Radiol* 2012;**4**:265–272.

33. Matsumoto H, Watanabe S, Kyo E, Tsuji T, Ando Y, Otaki Y, Cadet S, Slomka PJ, Berman DS, Dey D, Tamarappoo BK. Effect of tube potential and luminal contrast attenuation on atherosclerotic plaque attenuation by coronary CT angiography: In vivo comparison with intravascular ultrasound. *J Cardiovasc Comput Tomogr* 2019;**13**:219–225.

34. Chang H-J, Lin FY, Lee S-E, Andreini D, Bax J, Cademartiri F, Chinnaiyan K, Chow BJW, Conte E, Cury RC, Feuchtner G, Hadamitzky M, Kim Y-J, Leipsic J, Maffei E, Marques H, Plank F, Pontone G, Raff GL, Rosendael AR van, Villines TC, Weirich HG, Al’Aref SJ, Baskaran L, Cho I, Danad I, Han D, Heo R, Lee JH, Rivzi A, Stuijfzand WJ, Gransar H, Lu Y, Sung JM, Park H-B, Berman DS, Budoff MJ, Samady H, Shaw LJ, Stone PH, Virmani R, Narula J, Min JK. Coronary Atherosclerotic Precursors of Acute Coronary Syndromes. *J Am Coll Cardiol* 2018;**71**:2511–2522.

35. Budoff MJ, Bhatt DL, Kinninger A, Lakshmanan S, Muhlestein JB, Le VT, May HT, Shaikh K, Shekar C, Roy SK, Tayek J, Nelson JR. Effect of icosapent ethyl on progression of coronary atherosclerosis in patients with elevated triglycerides on statin therapy: final results of the EVAPORATE trial. *Eur Heart J* 2020;**41**:3925–3932.

36. Marwan M, Taher MA, El Meniawy K, Awadallah H, Pflederer T, Schuhbäck A, Ropers D, Daniel WG, Achenbach S. In vivo CT detection of lipid-rich coronary artery atherosclerotic plaques using quantitative histogram analysis: a head to head comparison with IVUS. *Atherosclerosis* 2011;**215**:110–115.

37. Matsumoto H, Watanabe S, Kyo E, Tsuji T, Ando Y, Otaki Y, Cadet S, Gransar H, Berman DS, Slomka P, Tamarappoo BK, Dey D. Standardized volumetric plaque quantification and characterization from coronary CT angiography: a head-to-head comparison with invasive intravascular ultrasound. *Eur Radiol* 2019;**29**:6129–6139.

38. Maurovich-Horvat P, Simon J. Tube Voltage or Luminal Attenuation?: Different Sides of the Same Coin. *JACC. Cardiovascular imaging*.

39. Achenbach S, Boehmer K, Pflederer T, Ropers D, Seltmann M, Lell M, Anders K, Kuettner A, Uder M, Daniel WG, Marwan M. Influence of slice thickness and reconstruction kernel on the computed tomographic attenuation of coronary atherosclerotic plaque. *J Cardiovasc Comput Tomogr* 2010;**4**:110–115.

40. Lee S-E, Sung JM, Andreini D, Al-Mallah MH, Budoff MJ, Cademartiri F, Chinnaiyan K, Choi JH, Chun EJ, Conte E, Gottlieb I, Hadamitzky M, Kim YJ, Lee BK, Leipsic JA, Maffei E, Marques H, Araújo Gonçalves P de, Pontone G, Shin S, Kitslaar PH, Reiber JHC, Stone PH, Samady H, Virmani R, Narula J, Berman DS, Shaw LJ, Bax JJ, Lin FY, Min JK, Chang H-J. Association Between Changes in Perivascular Adipose Tissue Density and Plaque Progression. *JACC Cardiovasc Imaging* 2022;**15**:1760–1767.

41. Cardoso R, Choi AD, Shiyovich A, Besser SA, Min JK, Earls J, PARADIGM Investigators, Blankstein R. How early can atherosclerosis be detected by coronary CT angiography? Insights from quantitative CT analysis of serial scans in the PARADIGM trial. *J Cardiovasc Comput Tomogr* 2023.

42. Williams MC, Golay SK, Hunter A, Weir-McCall JR, Mlynska L, Dweck MR, Uren NG, Reid JH, Lewis SC, Berry C, Beek EJR van, Roditi G, Newby DE, Mirsadraee S. Observer variability in the assessment of CT coronary angiography and coronary artery calcium score: substudy of the Scottish COmputed Tomography of the HEART (SCOT-HEART) trial. *Open Heart* 2015;**2**:e000234.

43. Park H-B, Arsanjani R, Sung JM, Heo R, Lee BK, Lin FY, Hadamitzky M, Kim Y-J, Conte E, Andreini D, Pontone G, Budoff MJ, Gottlieb I, Chun EJ, Cademartiri F, Maffei E, Marques H, Gonçalves P de A, Leipsic JA, Lee S-E, Shin S, Choi JH, Virmani R, Samady H, Chinnaiyan K, Stone PH, Berman DS, Narula J, Shaw LJ, Bax JJ, Min JK, Chang H-J. Impact of statins based on high-risk plaque features on coronary plaque progression in mild stenosis lesions: results from the PARADIGM study. *Eur Heart J Cardiovasc Imaging* 2023;**24**:1536–1543.

44. Omori H, Matsuo H, Fujimoto S, Sobue Y, Nozaki Y, Nakazawa G, Takahashi K, Osawa K, Okubo R, Kaneko U, Sato H, Kajiya T, Miyoshi T, Ichikawa K, Abe M, Kitagawa T, Ikenaga H, Saji M, Iguchi N, Ijichi T, Mikamo H, Kurata A, Moroi M, Iijima R, Malkasian S, Crabtree T, Min JK, Earls JP, Nakanishi R. Determination of lipid-rich plaques by artificial intelligence-enabled quantitative computed tomography using near-infrared spectroscopy as reference. *Atherosclerosis* 2023;**386**:117363.

45. Han D, Torii S, Yahagi K, Lin FY, Lee JH, Rizvi A, Gransar H, Park M-W, Roudsari HM, Stuijfzand WJ, Baskaran L, Ó Hartaigh B, Park H-B, Lee S-E, Ali Z, Kutys R, Chang H-J, Earls JP, Fowler D, Virmani R, Min JK. Quantitative measurement of lipid rich plaque by coronary computed tomography angiography: A correlation of histology in sudden cardiac death. *Atherosclerosis* 2018;**275**:426–433.

46. Schlett CL, Maurovich-Horvat P, Ferencik M, Alkadhi H, Stolzmann P, Scheffel H, Seifarth H, Nakano M, Do S, Vorpahl M, Kauczor H-U, Bamberg F, Tearney GJ, Virmani R, Hoffmann U. Histogram analysis of lipid-core plaques in coronary computed tomographic angiography: ex vivo validation against histology. *Invest Radiol* 2013;**48**:646–653.

47. Dey D, Cheng VY, Slomka PJ, Nakazato R, Ramesh A, Gurudevan S, Germano G, Berman DS. Automated 3-dimensional quantification of noncalcified and calcified coronary plaque from coronary CT angiography. *Journal of cardiovascular computed tomography*.

48. Williams MC, Kwiecinski J, Doris M, McElhinney P, D’Souza MS, Cadet S, Adamson PD, Moss AJ, Alam S, Hunter A, Shah ASV, Mills NL, Pawade T, Wang C, Weir McCall J, Bonnici-Mallia M, Murrills C, Roditi G, Beek EJR van, Shaw LJ, Nicol ED, Berman DS, Slomka PJ, Newby DE, Dweck MR, Dey D. Low-Attenuation Noncalcified Plaque on Coronary Computed Tomography Angiography Predicts Myocardial Infarction: Results From the Multicenter SCOT-HEART Trial (Scottish Computed Tomography of the HEART). *Circulation* 2020;**141**:1452–1462.

49. Graaf MA de, Broersen A, Kitslaar PH, Roos CJ, Dijkstra J, Lelieveldt BPF, Jukema JW, Schalij MJ, Delgado V, Bax JJ, Reiber JHC, Scholte AJ. Automatic quantification and characterization of coronary atherosclerosis with computed tomography coronary angiography: cross-correlation with intravascular ultrasound virtual histology. *Int J Cardiovasc Imaging* 2013;**29**:1177–1190.

50. Pérez de Isla L, Díaz-Díaz JL, Romero MJ, Muñiz-Grijalvo O, Mediavilla JD, Argüeso R, Andrés R de, Fuentes F, Sánchez Muñoz-Torrero JF, Rubio P, Álvarez-Baños P, Mañas D, Suárez Gutierrez L, Saltijeral Cerezo A, Mata P, SAFEHEART Investigators. Characteristics of Coronary Atherosclerosis Related to Plaque Burden Regression During Treatment With Alirocumab: The ARCHITECT Study. *Circ Cardiovasc Imaging* 2024;**17**:e016206.

51. Lin A, Manral N, McElhinney P, Killekar A, Matsumoto H, Kwiecinski J, Pieszko K, Razipour A, Grodecki K, Park C, Otaki Y, Doris M, Kwan AC, Han D, Kuronuma K, Flores Tomasino G, Tzolos E, Shanbhag A, Goeller M, Marwan M, Gransar H, Tamarappoo BK, Cadet S, Achenbach S, Nicholls SJ, Wong DT, Berman DS, Dweck M, Newby DE, Williams MC, Slomka PJ, Dey D. Deep learning-enabled coronary CT angiography for plaque and stenosis quantification and cardiac risk prediction: an international multicentre study. *Lancet Digit Health* 2022;**4**:e256–e265.

52. Buckler AJ, Gotto AM Jr, Rajeev A, Nicolaou A, Sakamoto A, St Pierre S, Phillips M, Virmani R, Villines TC. Atherosclerosis risk classification with computed tomography angiography: A radiologic-pathologic validation study. *Atherosclerosis* 2023;**366**:42–48.

53. Buckler AJ, Sakamoto A, Pierre SS, Virmani R, Budoff MJ. Virtual pathology: Reaching higher standards for noninvasive CTA tissue characterization capability by using histology as a truth standard. *Eur J Radiol* 2023;**159**:110686.

54. Mancini GBJ, Kamimura C, Yeoh E, Ryomoto A. Effects of adaptive or fixed thresholds and different platforms on the assessment of plaque characteristics using coronary computed tomography angiography. *J Cardiovasc Comput Tomogr* 2024.

55. Vaz S, Falkmer T, Passmore AE, Parsons R, Andreou P. The case for using the repeatability coefficient when calculating test-retest reliability. *PLoS One* 2013;**8**:e73990.

56. Bland JM, Altman DG. Statistical methods for assessing agreement between two methods of clinical measurement. *Lancet* 1986;**1**:307–310.

57. Beckerman H, Roebroeck ME, Lankhorst GJ, Becher JG, Bezemer PD, Verbeek AL. Smallest real difference, a link between reproducibility and responsiveness. *Qual Life Res* 2001;**10**:571–578.

58. Øvrehus KA, Dey D. Reply to: Reproducibility of semi-automatic coronary plaque quantification in coronary CT angiography with sub-mSv radiation dose; common mistakes. *J Cardiovasc Comput Tomogr* 2016;**10**:e23.

59. Cheng VY, Nakazato R, Dey D, Gurudevan S, Tabak J, Budoff MJ, Karlsberg RP, Min J, Berman DS. Reproducibility of coronary artery plaque volume and composition quantification by 64-detector row coronary computed tomographic angiography: an intraobserver, interobserver, and interscan variability study. *J Cardiovasc Comput Tomogr* 2009;**3**:312–320.

60. Schuhbaeck A, Dey D, Otaki Y, Slomka P, Kral BG, Achenbach S, Berman DS, Fishman EK, Lai S, Lai H. Interscan reproducibility of quantitative coronary plaque volume and composition from CT coronary angiography using an automated method. *Eur Radiol* 2014;**24**:2300–2308.

61. Øvrehus KA, Schuhbaeck A, Marwan M, Achenbach S, Nørgaard BL, Bøtker HE, Dey D. Reproducibility of semi-automatic coronary plaque quantification in coronary CT angiography with sub-mSv radiation dose. *J Cardiovasc Comput Tomogr* 2016;**10**:114–120.

62. Symons R, Morris JZ, Wu CO, Pourmorteza A, Ahlman MA, Lima JAC, Chen MY, Mallek M, Sandfort V, Bluemke DA. Coronary CT Angiography: Variability of CT Scanners and Readers in Measurement of Plaque Volume. *Radiology* 2016;**281**:737–748.

63. Lee S-E, Park H-B, Xuan D, Lee BK, Hong M-K, Jang Y, Chang H-J. Consistency of quantitative analysis of coronary computed tomography angiography. *J Cardiovasc Comput Tomogr* 2019;**13**:48–54.

64. Meah MN, Singh T, Williams MC, Dweck MR, Newby DE, Slomka P, Adamson PD, Moss AJ, Dey D. Reproducibility of quantitative plaque measurement in advanced coronary artery disease. *J Cardiovasc Comput Tomogr* 2021;**15**:333–338.

65. Flores Tomasino G, Han D, Pimentel R, Paz W, Liang J, Cheng VY, Slomka P, Berman DS, Dey D. Reproducibility of artificial intelligence-enabled plaque measurements between systolic and diastolic phases from coronary computed tomography angiography. *Eur Radiol* 2024.

66. Obuchowski NA, Buckler AJ. Estimating the Precision of Quantitative Imaging Biomarkers without Test-Retest Studies. *Acad Radiol* 2022;**29**:543–549.

67. Sheahan M, Ma X, Paik D, Obuchowski NA, St Pierre S, Newman WP 3rd, Rae G, Perlman ES, Rosol M, Keith JC Jr, Buckler AJ. Atherosclerotic Plaque Tissue: Noninvasive Quantitative Assessment of Characteristics with Software-aided Measurements from Conventional CT Angiography. *Radiology* 2018;**286**:622–631.

68. Flohr T, Schmidt B, Ulzheimer S, Alkadhi H. Cardiac imaging with photon counting CT. *Br J Radiol* 2023;**96**:20230407.

69. Zsarnoczay E, Fink N, Schoepf UJ, O’Doherty J, Allmendinger T, Hagenauer J, Wolf EV, Griffith JP 3rd, Maurovich-Horvat P, Varga-Szemes A, Emrich T. Ultra-high resolution photon-counting coronary CT angiography improves coronary stenosis quantification over a wide range of heart rates - A dynamic phantom study. *Eur J Radiol* 2023;**161**:110746.

70. Holmes TW, Yin Z, Stevens GM, Slavic S, Okerlund DR, Maltz JS, Pourmorteza A. Ultra-high-resolution spectral silicon-based photon-counting detector CT for coronary CT angiography: Initial results in a dynamic phantom. *J Cardiovasc Comput Tomogr* 2023;**17**:341–344.

71. Fink N, Zsarnoczay E, Schoepf UJ, O’Doherty J, Halfmann MC, Allmendinger T, Hagenauer J, Griffith JP 3rd, Vecsey-Nagy M, Pinos D, Ebersberger U, Ricke J, Varga-Szemes A, Emrich T. Impact of Cardiac Motion on coronary artery calcium scoring using a virtual non-iodine algorithm on photon-counting detector CT: a dynamic phantom study. *Int J Cardiovasc Imaging* 2023;**39**:2083–2092.

72. Wolf EV, Halfmann MC, Varga-Szemes A, Fink N, Kloeckner R, Bockius S, Allmendinger T, Hagenauer J, Koehler T, Kreitner K-F, Schoepf UJ, Münzel T, Düber C, Gori T, Yang Y, Hell MM, Emrich T. Photon-Counting Detector CT Virtual Monoenergetic Images for Coronary Artery Stenosis Quantification: Phantom and In Vivo Evaluation. *AJR Am J Roentgenol* 2024;**222**:e2330481.

73. Sartoretti T, McDermott MC, Stammen L, Martens B, Moser LJ, Jost G, Pietsch H, Gutjahr R, Nowak T, Schmidt B, Flohr TG, Wildberger JE, Alkadhi H. Tungsten-Based Contrast Agent for Photon-Counting Detector CT Angiography in Calcified Coronaries: Comparison to Iodine in a Cardiovascular Phantom. *Invest Radiol* 2024.

74. Grönberg F, Lundberg J, Sjölin M, Persson M, Bujila R, Bornefalk H, Almqvist H, Holmin S, Danielsson M. Feasibility of unconstrained three-material decomposition: imaging an excised human heart using a prototype silicon photon-counting CT detector. *Eur Radiol* 2020;**30**:5904–5912.

75. Koons EK, Rajiah PS, Thorne JE, Weber NM, Kasten HJ, Shanblatt ER, McCollough CH, Leng S. Coronary artery stenosis quantification in patients with dense calcifications using ultra-high-resolution photon-counting-detector computed tomography. *J Cardiovasc Comput Tomogr* 2024;**18**:56–61.

76. Koons EK, Gong H, Missert A, Chang S, Winfree T, Zhou Z, McCollough CH, Leng S. Learned high-resolution cardiac CT imaging from ultra-high-resolution PCD-CT. *Proc SPIE Int Soc Opt Eng* 2024;**12925**.

77. Halfmann MC, Bockius S, Emrich T, Hell M, Schoepf UJ, Laux GS, Kavermann L, Graafen D, Gori T, Yang Y, Klöckner R, Maurovich-Horvat P, Ricke J, Müller L, Varga-Szemes A, Fink N. Ultrahigh-Spatial-Resolution Photon-counting Detector CT Angiography of Coronary Artery Disease for Stenosis Assessment. *Radiology* 2024;**310**:e231956.

78. McCollough CH, Rajendran K, Leng S. Standardization and Quantitative Imaging With Photon-Counting Detector CT. *Invest Radiol* 2023;**58**:451–458.

79. Danielsson M, Persson M, Sjölin M. Photon-counting x-ray detectors for CT. *Phys Med Biol* 2021;**66**:03TR01.

80. Fleiter TR. Editorial Comment: Photon-Counting Detector CT—The New Era of Routine Quantitative Imaging? *American Journal of Roentgenology* 2023;**221**:547–547.

81. Salyapongse AM, Rose SD, Pickhardt PJ, Lubner MG, Toia GV, Bujila R, Yin Z, Slavic S, Szczykutowicz TP. CT Number Accuracy and Association With Object Size: A Phantom Study Comparing Energy-Integrating Detector CT and Deep Silicon Photon-Counting Detector CT. *AJR Am J Roentgenol* 2023;**221**:539–547.

82. Seltzer SM. XCOM: Photon Cross Sections Database | NIST. 2009.

83. Cropp RJ, Seslija P, Tso D, Thakur Y. Scanner and kVp dependence of measured CT numbers in the ACR CT phantom. *J Appl Clin Med Phys* 2013;**14**:4417.

84. Nakajima S, Ito H, Mitsuhashi T, Kubo Y, Matsui K, Tanaka I, Fukui R, Omori H, Nakaoka T, Sakura H, Ueno E, Machida H. Clinical application of effective atomic number for classifying non-calcified coronary plaques by dual-energy computed tomography. *Atherosclerosis* 2017;**261**:138–143.

85. Machida H, Tanaka I, Fukui R, Shen Y, Ishikawa T, Tate E, Ueno E. Dual-Energy Spectral CT: Various Clinical Vascular Applications. *Radiographics* 2016;**36**:1215–1232.

86. Bluemke DA, Lima JAC. Cardiac Imaging 2040. *Radiology* 2023;**307**:e230160.

87. Silicon Dreams: A Technological Breakthrough to Let Doctors Peer Inside the Body with Startling Clarityhttps://www.gehealthcare.co.uk/insights/article/silicon-dreams-a-technological-breakthrough-to-let-doctors-peer-inside-the-body-with-startling-clarity (March 24, 2024)

88. Schmidt TG, Sidky EY, Pan X, Barber RF, Grönberg F, Sjölin M, Danielsson M. Constrained one-step material decomposition reconstruction of head CT data from a silicon photon-counting prototype. *Med Phys* 2023;**50**:6008–6021.

89. Silva J da, Grönberg F, Cederström B, Persson M, Sjölin M, Alagic Z, Bujila R, Danielsson M. Resolution characterization of a silicon-based, photon-counting computed tomography prototype capable of patient scanning. *J Med Imaging (Bellingham)* 2019;**6**:043502.
